# Intermediate filament IFFO1 negatively regulates the migration of lung cancer cells by inhibiting the IQGAP3-Cdc42 interaction

**DOI:** 10.1038/s41419-025-07846-z

**Published:** 2025-07-10

**Authors:** Yuanling Ye, Fan Shen, Maoli Yan, Conghui Zhan, Jiahua Lv, Mengqin Zou, Zhifeng Wang, Shaokai Ning, Yanfei Gao, Jingxian Wu, Wen Li

**Affiliations:** 1 Center for Medical Epigenetics, School of Basic Medical Sciences, Chongqing Medical University, Chongqing, China; 2 Department of Urology, Henan Provincial People’s Hospital, Zhengzhou University People’s Hospital, Henan University People’s Hospital, Zhengzhou, Henan Province China; 3 Wuxi Maternal and Child Health Hospital, Wuxi School of Medicine, Jiangnan University, Jiangsu, China; 4 Department of pathology, College of Basic Medicine of Chongqing Medical University, Chongqing, China; 5 Molecular Medicine Diagnostic and Testing Center, Chongqing Medical University, Chongqing, China; 6 Department of Pathology, the First Affiliated Hospital of Chongqing Medical University, Chongqing, China

**Keywords:** Cytoskeleton, Cell migration

## Abstract

The metastasis of lung cancer represents a significant factor contributing to the failure of clinical treatment, and the mechanisms involved are intricate and not yet completely elucidated. Intermediate filaments, which constitute a key element of the cytoskeleton, function not only as diagnostic markers but also play a role in malignant processes, including tumor proliferation, apoptosis, and migration. The intermediate filament IFFO1 is essential for genomic stability, and its expression level serves as a prognostic indicator in various tumors. However, the functional contributions of IFFO1 in tumor progression remain inadequately understood. Our study revealed that IFFO1 is downregulated in lung cancer patients and is correlated with a poorer prognosis. The depletion of IFFO1 led to enhanced tumor proliferation both in vitro and in vivo, as well as increased cellular migration, alterations in the cytoskeleton and modifications in GTPase-mediated signal transduction. Mechanically, IFFO1 was found to interact directly with the GTPase-related scaffold IQGAP3 through the 2 A coiled-coil domain, which was critical for the inhibition of cellular mobility. Moreover, the increased cell migration observed in IFFO1-deficient cells was substantiated as being facilitated by IQGAP3. Finally, IFFO1 was shown to inhibit the association of IQGAP3 with its effector Cdc42 in a dose-dependent manner. Our study demonstrates the multifaceted roles of IFFO1 in lung cancer by modulating the IQGAP3-Cdc42 axis and suggests the potential for identifying tumor targets through the examination of intermediate filament networks and their associated co-factors.

## Introduction

Intermediate filaments (IFs) exhibit low solubility and high flexibility due to their coiled-coil structure, and the identification of their binding cofactors is essential for the investigation of their diverse functions [1]. The size of IFs lies between those of actin and microtubules, and IFs interact with the cytoskeleton to form a flexible network [2]. Metastatic tumor cells are associated with considerable cytoskeleton remodeling and a dynamic equilibrium between adhesion and de-adhesion to the surrounding matrix [3]. In the context of tumor pathology, notable variations are observed in the effect of specific IFs and related adhesion signals on cell migration [4]. The truncated form of keratin promotes cell metastasis by affecting the structure of desmosomes and cell-cell adhesion [5]. The interaction between epithelial protein keratin and mesenchymal protein vimentin enhanced cancer progression [6]. Because of the presence of a certain degree of redundancy and compensation among multiple IFs, deciphering the precise function and subtle regulatory patterns is crucial for identifying potential predictive or therapeutic targets.

The intermediate filament IFFO1 participates in the non-homologous end joining repair and anchors the broken DNA ends onto the nucleoskeleton, thereby suppressing chromosomal translocation [7]. IFFO1 is differentially expressed in tumor and control tissues in liver cancer [8], and is downregulated at both transcriptional and post-transcriptional levels in ovarian cancer with the inhibition of tumor metastasis and reversing drug resistance [9]. In studies involving ascites and blood samples for ovarian cancer, promoter methylation of the IFFO1 exhibited potential as an effective biomarker [10, 11]. These findings suggest that IFFO1 may function as a tumor suppressor, while it remains unclear whether IFFO1 perceives external signals and plays an independent and composite role.

The IQ motif-containing GTPase-activating protein 3 (IQGAP3) belongs to an evolutionarily conserved family of GTPase-activating proteins (IQGAP1, IQGAP2, and IQGAP3) [12], and play a role in cytoskeleton regulation, focal adhesion, Ca^2+^ channel transport, and G protein signaling through different binding partners [13, 14]. IQGAP3 and IQGAP1 activate ERK-related cell proliferation and function as oncogenes, while IQGAP2 tends to inhibit tumor progression [14, 15]. IQGAP3 promotes microvascular invasion in liver cancer, which depends on the maintenance of GTPase Rac1 activity [16]. The RasGAP-related domain (GRD) of IQGAP3 is the most critical component for its interaction with the small Rho GTPase Cdc42 [15]. A large-scale sequencing of primary and metastatic lung adenocarcinoma cells indicated that IQGAP3 is a potential marker of disease progression and poor prognosis [17]. IQGAPs and their effector Cdc42 serve as crucial mediators in microtubule crosstalk and actin filament dynamics. However, a more comprehensive investigation is necessary to elucidate the temporal and spatial regulation of the recruitment of other regulatory proteins, particularly those associated with the more adaptable IFs in cytoskeleton-related physiological processes.

In our study, we comprehensively investigated the significance of IFFO1, whose function remains unelucidated in lung cancer, with a specific focus on the interaction domains that regulate tumor progression. Our results indicated that IFFO1 was downregulated in lung cancer and its subtypes, and this downregulation was strongly correlated with tumor progression and recurrence. *IFFO1*^*-/-*^ cells exhibited accelerated proliferation, particularly in three-dimensional (3D) cultured cell spheres, as well as increased cell movement, leading to enhanced migration. Significantly, the phenotypes of *IFFO1*^*-/-*^ cells were reversed upon the re-expression of the full-length IFFO1. Additionally, IFFO1 directly interacts with IQGAP3 through the 2 A coiled-coil domain. This interaction is essential for the attenuation of the PI3K-Akt signaling pathway, the inhibition of the interaction between IQGAP3 and Cdc42, and the suppression of cellular mobility. This study indicated that IFFO1 functions not only as an intracellular signaling molecule, but also plays a pivotal role in regulating the mechanisms that govern tumor cell migration through the coordination of protein-protein interactions. These findings offer valuable insights for the accurate diagnosis of tumors and the identification of therapeutic targets related to intermediate filaments.

## Materials and methods

### Clinical specimens and tissue microarray

This study was approved by the Ethics Committee of Chongqing Medical University (Number: 2024036) and followed the Declaration of Helsinki. The slices of tumor tissue were taken from the Department of Pathology at Chongqing Medical University and obtained informed consent from the subjects. The patients’ information was listed in Supplementary Table 1. A tissue microarray containing lung cancer and adjacent tissue samples was obtained from Shanghai Outdo Biotech (Cat No. HLugA180Su08, Shanghai, China). IFFO1 expression was determined by immunohistochemical staining with appropriate antibodies and compared between tumor tissues and adjacent normal tissues.

### Data sources and bioinformatic analysis

The RNA-seq data were acquired from the TCGA (https://portal.gdc.cancer.gov/) and GEO (https://www.ncbi.nlm.nih.gov/geo/) databases, and the obtained data were standardized and log2 transformed. Dataset normalization and background noise removal were conducted with the affy package in R. Gene ontology (GO) and Kyoto Encyclopedia of Genes and Genomes (KEGG) were performed for functional enrichment analyses of hub genes.

### Survival analysis

Patients were assigned to high- and low-expression groups based on the median expression of IFFO1 and IQGAP3. The Kaplan–Meier survival curves were used to evaluate overall survival. The log-rank test was utilized to calculate p-values, and hazard ratios (HRs) were calculated together with their corresponding 95% confidence intervals (95% CIs).

### Cell culture and transfection

NCI-H1299, NCI-H358, Calu-1 and NCI-H292 cell lines are obtained from Pricella Biotechnology. MRC5 and A549 cells were provided by Youquan Bu’s lab in Chongqing Medical University. NCI-H1299 and NCI-H358 cells were cultured in RPMI-1640 medium (Pricella, #PM150110). NCI-H292, MRC5, HEK-293T and A549 cells were cultured in high-glucose DMEM medium (Pricella, #PM150210). Calu-1 cells were cultured in McCoy’s 5 A medium (Pricella, #PM150710). All cell cultures were supplemented with 10% fetal bovine serum (FBS) (Excell, #FSP500) and 1% penicillin/streptomycin (10,000 µg/mL, Gibco, #15140-122) and cultured at 37 °C in a 5% CO_2_ environment. All cells were tested for *Mycoplasma* contamination every month and were ensured to be contamination-free. Polyethyleneimine (PEI, Yeasen Biotechnology) and jetPRIME transfection reagent (Polyplus, #101000015) were used for plasmid transfection in HEK-293T and other cells, respectively.

### Plasmids

Full-length IFFO1 and domain-deletion mutants were subcloned into the pCI-neo vector. Full-length IFFO1 and other domain deletion mutants were subcloned into the pCMV-C-mCherry vector (a gift from Xudong Duan’s lab in Chongqing Medical University). For IQGAP3 expression, pCDNA3.1-IQGAP3 was obtained from BGI’s Gene Synthesis. The lentivirus vectors were provided by Dongyi Xu’s lab in Peking University.

### The shRNA and siRNA transfections

HEK-293T cells were transfected with short hairpin RNA (shRNA) and packing plasmids using PEI. The lentivirus was harvested from the cell supernatant and then infected into NCI-H1299 cells. The shRNAs used targeting IFFO1 were: CGAGTACAAGCGGAGATGCTT and GCCTGGCTTGTCGTGGGTGCA. The shRNAs used targeting Lamin A/C were: GAAGCAACTTCAGGATGAGAT and GCCGTGCTTCCTCTCACTCAT. For siRNA transfections, the Lipofectamine RNAi MAX transfection reagent (ThermoFisher, 13778075) was utilized following the manufacturer’s instructions. The siRNAs targeting IQGAP3 were: CGUCCGAACUGGCCAAAUA and GGGUGUGGCUGUCAUGAAA.

### Generation of *IFFO1* knockout cells

The guide sequences targeting IFFO1 (GCCTGCTGCTCCTGCTGCAGG and GTGGCTTGTCGTGGGTGCACC) were constructed in the pX330 plasmid [18], followed by transfection of the plasmids into cells. After 10–14 days of cultivation, single colonies were selected. The relevant DNA fragments were amplified by PCR and sequenced using the following primers: GGCTCTTCTGGCTCGCCTTCCATC/CGGCGAAGTGGTCGCCTCCCAGTG and CCCGGCACCATCTGGTCGTTCTC/ GTGAGTGGTATGACACAGAGAGAC.

### Mice xenograft models

All animal procedures to be employed in the project were approved by Institutional Animal Care and Use of Chongqing Medical University (IACUC-CQMU-2024-0266). Considering the tumorigenicity of different cell lines and the ethical principle of reducing animal usage, sixteen (NCI-H1299 cell line) and ten (NCI-H358 cell line) male BALB/c nude mice at 8 weeks of age were randomly divided into two groups to establish tumor xenografts. Wild-type and *IFFO1*^−/−^ cells were resuspended in 50 µL PBS at the density of 2 × 10^6^ cells (for NCI-H1299) or 5 × 10^6^ cells (for NCI-H358) per mouse and mixed with an equal volume of Matrigel (Corning, #354234). The weight and tumor size of mice were monitored, and the tumor volume was calculated according to the following equation: volume = length × (width)^2^/2. When measuring tumor size, two researchers conducted a double-blind assessment.

### In vivo tumor metastatic assay

Considering the tumorigenicity of different cell lines and the ethical principle of reducing animal usage, twelve male BALB/c nude mice at 8 weeks of age were randomly divided into two groups for each cell line. Wild-type and *IFFO1*^−/−^ NCI-H1299 (1 × 10^6^ cells) and NCI-H358 (2 × 10^6^ cells) cells were injected via tail vein into male BALB/c nude mice aged 6 to 8 weeks to perform in vivo tumor metastasis assays. Following eight to ten weeks post-injection, lung metastasis was evaluated through histopathological analysis. All animal procedures were approved by Institutional Animal Care and Use of Chongqing Medical University (IACUC-CQMU-2024-0266). When measuring tumor size, two researchers conducted a double-blind assessment.

### Cell proliferation assay

NCI-H1299 and NCI-H358 cells were plated in 96-well plates at the density of 1 × 10^3^ cells/well and cultured at 37 °C in a 5% CO_2_ environment. Metabolically active cells were determined with the Cell Counting Kit-8 (CCK8, MedChemExpress) every day for 1 week in accordance with the manufacturer’s instructions. These experiments were independently repeated at least three times under the same conditions.

### The 3D cell culture

Matrigel (Corning, #354234) was thawed slowly on ice and mixed with suspended cells (1 × 10^3^ cells) at different concentrations. The Matrigel/cell mixture was evenly spread on a 12-well plate and solidified at 37 °C in a 5% CO_2_ environment for 30 min. After a specific cultivation period, the 3D growth of cells was observed using an inverted phase contrast microscope (Olympus, Tokyo, Japan), and the size of cell spheres was calculated.

### Immunohistochemistry

Fresh tissues were fixed with 4% paraformaldehyde solution overnight and embedded in paraffin. Subsequently, 4 µm-thick paraffin-embedded sections were prepared and dewaxed with xylene, followed by rehydration with a graded series of 100% to 75% ethanol. The rehydrated tissue sections were incubated with 0.3% H_2_O_2_, followed by blocking with 5% bovine serum albumin (BSA)/PBS for 30 min. The slides were incubated with primary antibodies for IFFO1 overnight at 4 °C and further incubated with HRP-conjugated secondary antibodies for 30 min at room temperature. Proteins were visualized by DAB, and images were acquired with a bright field microscope (KEYENCE BZ-X800E). TUNEL immunostaining of paraffin-embedded sections was performed in accordance with the manufacturer’s instructions (Beyotime, #C1086). The IFFO1 expression levels within tumors were categorized based on staining intensity into the following grades: negative (level score = 0), weak brown (level score = 1), moderate brown (level score = 2), and strong brown (level score = 3). Furthermore, the proportion of stained cells was classified into five intensity levels: 0–3% (intensity score = 0), 3–25% (intensity score = 1), 26–50% (intensity score = 2), 51–75% (intensity score = 3), and 76–100% (intensity score = 4). The final immunoreactivity score (IRS) = intensity score × level score [19, 20].

### H&E staining

The mouse lung tissues were fixed in 4% paraformaldehyde for at least 24 h. Following the embedding of the tissue in paraffin and subsequent sectioning, the tumor samples were stained using Hematoxylin and Eosin (H&E).

### Western blotting

Cells were collected with a disposable cell scraper, and total protein was extracted using RIPA lysis buffer (1% Triton X-100, 1% sodium deoxycholate, 0.1% SDS). The extracted proteins were separated by sodium dodecyl sulfate-polyacrylamide gel electrophoresis (Yazyme, #PG111 and #PG112), and then transferred to a polyvinylidene fluoride membrane (Millipore, #IPVH00010). The membranes were blocked with PBST (PBS with 0.1% Tween-20) containing 5% skimmed milk, followed by incubation with primary antibodies at 4 °C overnight and with the secondary antibodies at room temperature for 2 h. The obtained signals were quantified using the automatic chemiluminescence gel imaging system (BIO-RAD, CANICES-3(A)/NMB-3(A)). Full and uncropped blots are provided in the Supplementary materials.

### Antibodies

Antibodies against Phospho-p44/42 MAPK(Erk1/2) (Thr202/Tyr204) (#4370), N Cadherin (#P19022), Phospho-Akt (Ser473) (#9271), and p44/42 MAPK (Erk1/2) (#4695) were purchased from Cell Signaling Technology. Antibodies against IFFO1 (ab242130) were purchased from Abcam. Antibodies against Anti-DDDDK Tag mAb (#M185-3L) were purchased from MBL BEIJING BIOTECH CO, LTD (Beijing, China). Antibodies against Lamin A/C (#P48678) and N Cadherin (#GB12135) were purchased from Servicebio (Wuhan, China). Antibodies against GAPDH (#60004-1-Ig), MMP1 (#10371-2-AP), MMP2 (#10373-2-AP), MMP9 (#10375-2-AP), IQGAP3 (#25930-1-AP), XRCC4 (#15817-1-AP), CCAR2 (#22638-1-AP), MYC tag (#16286-1-AP), Vimentin (#10366-1-AP), Caspase 9/p35/p10 (#10380-1-AP), Caspase-3 /p17/p19 (#19677-1-AP), BAX (#50599-2-Ig), Beta Actin (#66009-1-Ig), Cdc42 (#10155-1-AP), HSP90 (#13171-1-AP), PI3 Kinase p110 Beta (#20584-1-AP), AKT (#10176-2-AP), Beta Tubulin (#10094-1-AP) and IFFO1 (#16041-1-AP) were purchased from Proteintech (Wuhan, China).Anti-mouse IgG HRP-linked antibody (#7076P2) and anti-rabbit IgG HRP-linked antibody (#7074P2) were purchased from Cell Signaling Technology.

### Immunofluorescence

Cells were first seeded on sterile glass slides (Servicebio, #WG655) and cultured for 24 h. After being fixed with 3% paraformaldehyde for 10 min at room temperature, the cells were incubated with PBST containing 0.5% Triton X-100 and washed three times with PBST. The cells were then blocked with PBS containing 5% BSA, incubated with primary antibodies at 4 °C overnight, further incubated with secondary antibodies at room temperature for 1 h, and finally mounted with the Antifade Mounting Medium (Beyotime, #P0131). The coverslips were examined under a Leica TCS SP8 microscope with an ×63 plan apo oil immersion objective lens.

### Transwell assay

A total of 2 × 10^4^ cells were mixed with a culture medium containing 1% FBS and seeded onto the upper Transwell chamber (FALCON, #353097; pore size: 0.8 µm), and the entire chamber was placed in a 24-well plate with 700 µL medium containing 10% FBS. After incubation for 24 h, the cells at the bottom of the chamber were fixed and stained with Crystal Violet. Nine fields were randomly selected under the Nikon eclipse Ts2R microscope with an x10 objective lens. These experiments were independently repeated at least three times under the same conditions. In order to mitigate the influence of cell proliferation on migration, the cells were subjected to treatment with 2 µg/ml Mitomycin C (MMC) for 2 h before examined by the Transwell assay.

### Live-cell time-lapse imaging

Cells were placed in a glass-bottomed cell culture dish (CORNING, #D35C4-20-1-N). Images were taken by the KEYENCE BZ-X800E integrated fluorescence microscope. Live cell videos were analyzed by ImageJ software with the TrackMate plug-in for cell path tracing [21]. Cells were selected to determine the average velocity and total distance, and the dividing and adhering cells were excluded under the following conditions: LoG detector (estimated object diameter: 0.402-inch, quality threshold: 0.42) and advanced Kalman Tracker (initial search radius: 1.0-inch, search radius: 0.5-inch, Max Frame Gap: 1 frame).

### Phalloidin stain

The cytoskeleton was stained with phalloidin (Yeasen Biotechnology, #40734ES75) in accordance with the manufacturer’s instructions. Briefly, cells were seeded on sterile glass slides and fixed with 3% formaldehyde at room temperature for 10 min. After washing with PBS, the cells were permeabilized with PBS containing 0.5% Triton X-100 for 5 min. TRITC phalloidin (100 nM; Yeasen Biotechnology, #40734ES75) was applied and mounted with the Antifade Mounting Medium (Beyotime, #P0131). Fluorescence was observed under a confocal microscope (Leica TCS SP8) and STEDYCON super-resolution microscope (Nikon) with TRITC excitation/emission filters (Ex/Em = 545/570 nm) and x100 objective lens.

### RNA isolation and real-time PCR

Total RNA was isolated from cells with TRIZOL (Invitrogen, #15596026). The RT Master Mix (Takara, #RR047A) was used to synthesize cDNA. Real-time PCR was conducted with SYBR Green Master Mix (Yeasen Biotechnology, # 11201) in accordance with the manufacturer’s instruction. The mRNA levels of each gene were normalized to those of GAPDH mRNA. Primers for COL6A2 were: CGTGGAGACTCAGGACAGCCA and CCTTTCAAGCCAAAGTCGCCTC. Primers for ZEB1 were: GATGATGAATGCGAGTCAGATGC and ACAGCAGTGTCTTGTTGTTGT. Primers for JAK3 were: AGTGACCCTCACTTCCTGCTGT and GGCTGAACCAAGGATGATGTGG. Primers for ITGB7 were: ATCGAGGACAGTGCAACCACGT and TCAGCTCCTCTGAGAAGCCAAG. Primers for Cdc42 were: TGACAGATTACGACCGCTGAGTT and GGAGTCTTTGGACAGTGGTGAG. Primers for PIK3CG were: GAGAGCTTGGAGGACGATGATG and CCACGCTTCAGCAGAAATCTGG. Primers for COL4A1 were: TGTTGACGGCTTACCTGGAGAC and GGTAGACCAACTCCAGGCTCTC. Primers for ZO-1 were: GTCCAGAATCTCGGAAAAGTGCC and CTTTCAGCGCACCATACCAACC. Primers for FGF1 were: TGCACAGCGTGCGGTACCTCT and CGGTACACATTGTAGCCATCTGG. Primers for FZD7 were: GTCTTCAGCGTGCTCTACACAG and ACGGCATAGCTCTTGCACGTCT. Primers for WNT16 were: TCGGAAACACCACGGGCAAAGA and GCGGCAGTCTACTGACATCAAC. Primers for SMAD7 were: TGTCCAGATGCTGTGCCTTCCT and CTCGTCTTCTCCTCCCAGTATG. Primers for COL4A6 were: TGGCATCAAGGGCAAATCTGGG and ATCCAGGAGGACCTTTCTTGCC. Primers for SEMA6D were: GCATCTCGTGACCCGTATTGTG and CCTAGATGAGCTGTGTTGCCGA. Primers for SEMA6A were: ACCTGTATTGCCTCCAGAGACC and CCAGACCATCTGTATTGCCACG. Primers for ICAM1 were: AGCGGCTGACGTGTGCAGTAAT and TCTGAGACCTCTGGCTTCGTCA. Primers for PDGFA were: CAGCGACTCCTGGAGATAGACT and CGATGCTTCTCTTCCTCCGAATG. Primers for HTR7 were: TCATGACCCTGTGCGTGATCAG and GACGGAGAGAATCATCTTCGCC. Primers for GAPDH were: GACAGTCAGCCGCATCTTCT and GCGCCCAATACGACCAAATC. Each gene was independently repeated at least three times under the same conditions.

### Immunoprecipitation

Cells between 48 and 72 h of transfection were collected, pre-cooled, washed with PBS, lysed using NTEN buffer (20 mM Tris-HCl (pH 7.5), 10% glycerol, 150 mM NaCl, 0.5% NP40, 1 mM PMSF, 1 µg/mL leupeptin, and 1 µg/mL aprotinin), and sonicated of 10 s on/20 s off for three cycles by Qsonica sonicator (Q800R3). Following centrifugation at 14,000 rpm at 4 °C for 30 min, 50 µL of the supernatant was collected to prepare the input sample. The remaining supernatant was incubated overnight with 30 µL anti-DYKDDDDK (Flag) affinity gel (Yeasen Biotechnology, #20584). After washing with washing buffer (20 mM Tis- HCl, pH7.5, 150 mM NaCl, 10% Glycerol, 1% Nonidet P40 Substitute, 5 mM MgCl2, 2 mM EDTA, pH 8.0), the protein samples were denatured in 1 × loading buffer.

### RNA sequencing

Total RNA was extracted using Trizol (Invitrogen, #15596026), and precise quantification was performed using NanoDrop One/OneC, along with RNA quality assessment by Agilent 4200 TapeStation. Library construction and sequencing were performed by the Illumina PE150 platform at HaploX Biotechnology (Shenzhen, China).

### Mass spectrometry

The identification of protein samples is performed at BGI (Shenzhen, China). The protein samples underwent enzymatic digestion, which was subsequently followed by high-performance liquid chromatography (UltiMate 3000 UHPLC, Thermo Fisher Scientific) coupled with tandem mass spectrometry (Orbitrap Fusion Lumos, Thermo Fisher Scientific) for detection.

### Apoptosis detection

Apoptosis detection was performed using an Annexin V-FITC Apoptosis Detection Kit (Beyotime, C1062S) according to the manufacturer’s instructions. Briefly, Annexin V-FITC and propidium iodide (PI) are introduced for incubation following the collection and washing of the cells. Thereafter, the cells are subjected to analysis for scattering and fluorescence utilizing a flow cytometer (cytoflex).

### Active Ras pull-down assay

The GTP-bound active forms of Ras were pull-down in cell lysate with IFFO1 knockout and overexpression using the Active Ras Pull-Down and Detection Kit (Thermo Fisher, #16117) according to the manufacturer’s instructions. Briefly, the glutathione resin combined with GST-Raf1-RBD was incubated with the cell lysate, followed by washing and elution. The eluted samples were then separated by SDS-PAGE and detected using anti-Ras antibodies.

### GST-pull down assay

IFFO1 coding sequence was constructed into the recombinant plasmid expression vector pGEX-4T-1, followed by transformed into E. coli BL-21 cells. The cells were grown until an OD 600 = 0.8 and induced with 0.5 mM isopropylthiogalactoside (IPTG) at 25 °C for 6 h. The cell pellet was lysed in NTEN buffer, and the supernatant was incubated with Glutathione Sepharose 4B beads (GE Healthcare) at 4 °C for 6 h. The beads were washed twice with wash buffer (20 mM Tris-HCl (pH 7.5), 150 mM NaCl, 5% glycerol, 0.1% NP40, 1 mM PMSF). 10 μg of pCDNA3.1-IQGAP3 and its truncated forms were transfected into HEK-293T cells using polyethyleneimine. Cells were harvested after 3 days and lysed in NTEN buffer, followed by incubation with IFFO1-bound Glutathione Sepharose beads at 4 °C for 3 h. The beads were washed with the above wash buffer four times, and the protein samples were denatured in 1 × loading buffer.

### Statistical analysis and reproducibility

Data analyses were performed with GraphPad Prism 9 (GraphPad Software, La Jolla, CA). The experiments were independently repeated at least three times under the same conditions. All immunoblots were performed at least three times. Data were analyzed with Student’s *t*-test and expressed as mean ± SD. Statistical significance was determined as follows: ****p < 0.0001; ***p < 0.001; **p < 0.01; *p < 0.05; not significant (ns) *p* > 0.05.

## Results

### IFFO1 depletion promotes tumor growth

**Fig. 1:**
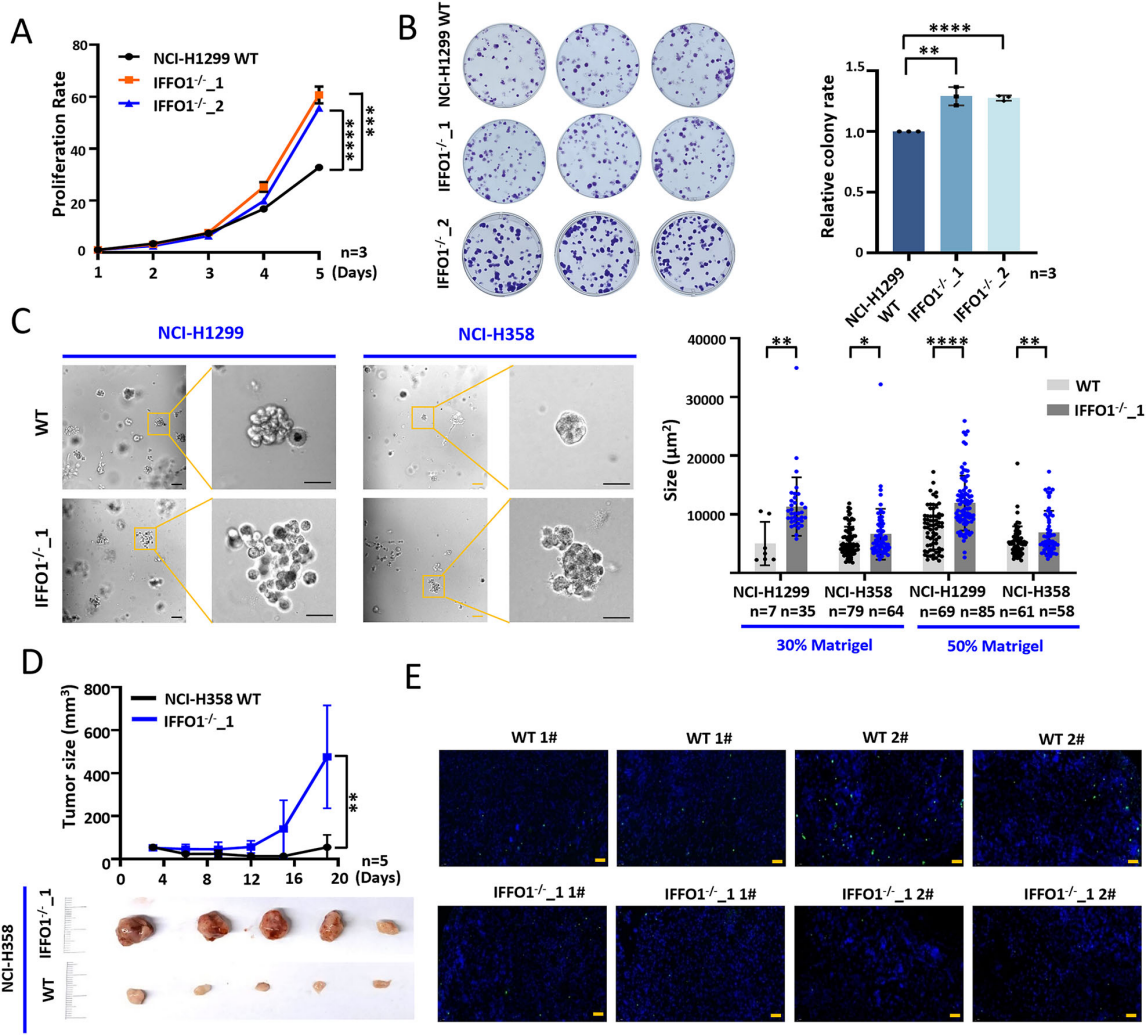
IFFO1 depletion promotes tumor growth. **A** Cell proliferation in wild-type (WT) and *IFFO1*^*-/-*^ NCI-H1299 cells. **B** Clone formation and statistical results after *IFFO1* depletion in NCI-H1299 cells. The data obtained are the result of at least three independent replicates and normalized with WT cells. **C** Spheres in NCI-H1299 and NCI-H358 cells cultured in three-dimensional Matrigel (left). Size of spheres of the two cell lines cultured at 30% and 50% concentration of Matrigel for 7 days (right). Scale bar = 50 μm. **D** Tumor xenografts (down) and growth curve (up) in BALB/c nude mice with NCI-H358 cells after 20 days of transplantation. **E** TUNEL staining of mouse xenografts derived from NCI-H358. Blue, DAPI. Green, TUNEL. Scale bar = 100 μm. *p < 0.05. **p < 0.01. ***p < 0.001. ****p < 0.0001.

To investigate the function and mechanisms of IFFO1 in lung cancer, IFFO1 expression was assessed in several cell lines. The result indicated a reduced expression of IFFO1 in lung cancer cell lines compared to human embryonic lung fibroblast cell line MRC-5 (Supplementary Fig. 1A). The CRISPR/Cas9 knockout was performed targeting *IFFO1* in NCI-H1299 and NCI-H358 cells, and the knockout clones were obtained (Supplementary Fig. 1B). *IFFO1*^−/−^ cells exhibited enhanced proliferation in both NCI-H1299 and NCI-H358 cells (Fig. 1A, Supplementary Fig. 1C), as well as a significant increase in clone formation (Fig. 1B). The 3D cell culture based on Matrigel can simulate the structure and complex functions of a tissue, and the cell spheroids exposed to a nutrient-rich medium can better reflect cell proliferation ability [22]. The proliferation of *IFFO1*^−/−^ cells cultured in 3D Matrigel was markedly increased, and this observation remains consistent regardless of the Matrigel concentration utilized (Fig. 1C). Meanwhile, the deletion of IFFO1 does not exert a notable impact on cell apoptosis (Supplementary Fig. 1D). *IFFO1*^−/−^ cells were subcutaneously implanted in BALB/c nude mice, and the tumor volume of *IFFO1*^−/−^ cells in transplanted mice was significantly larger than that of wild-type cells (Fig. 1D, Supplementary Fig. 1E, F). *IFFO1*^−/−^ xenograft exhibited decreased apoptosis (Fig. 1E, Supplementary Fig. 1G). These results indicated that IFFO1 inhibited the proliferation of lung cancer cells both in vivo and in vitro.

### IFFO1 depletion increases lung cancer cell migration

**Fig. 2:**
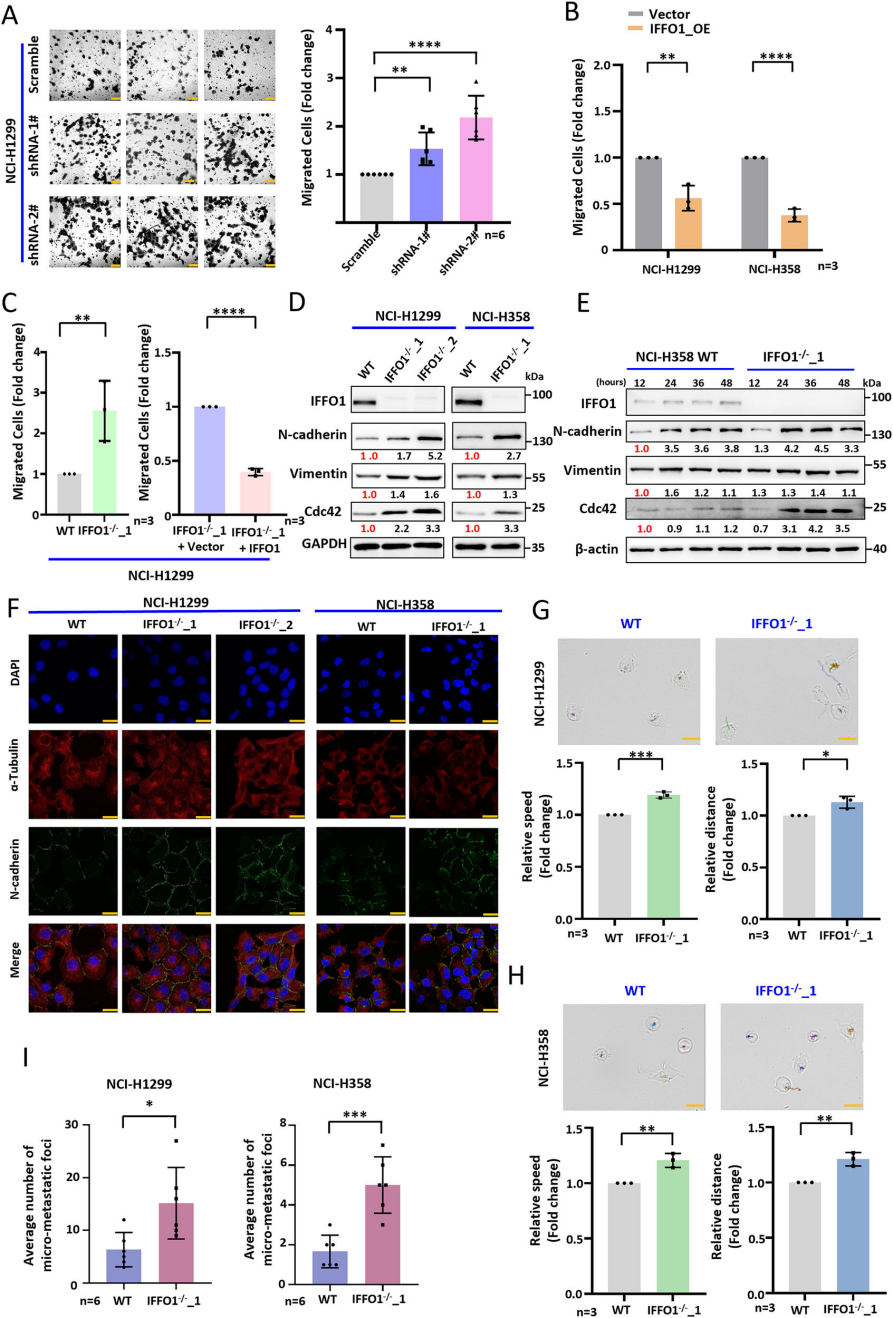
IFFO1 depletion increases lung cancer cell migration. **A** Migration of IFFO1-depleted NCI-H1299 cells. Left, migrated cells detected by Transwell assay. Scale bar = 100 μm. Right, statistical analysis of migrated cells. Transwell assays were performed in IFFO1 over-expression (OE) in WT cells (**B**), *IFFO1*^−/−^ NCI-H1299 cells, and IFFO1 re-expression in *IFFO1*^−/−^ NCI-H1299 cells (**C**). **D** Western blotting to determine migration markers. The relative value of protein expression was obtained by comparing with GAPDH and normalizing with WT cells. **E** Western blotting of migration markers in WT and *IFFO1*^−/−^ NCI-H358 cells over time. The relative value of protein expression was obtained by comparing with β-actin and normalizing with samples in 12 h. **F** Immunofluorescence staining of N-cadherin. Scale bar = 30 μm. Single cell movement trajectory and the average speed and total distance recorded during 175 min in NCI-H1299 (**G**) and NCI-H358 cells (**H**). Scale bar = 30 μm. **I** Pulmonary micro-metastases following the injection of tumor cells via the tail vein in nude mice over a duration of 8 to 10 weeks. *p < 0.05. **p < 0.01. ***p < 0.001. ****p < 0.0001.

Lentivirus packaged with various shRNAs significantly inhibited IFFO1 expression (Supplementary Fig. 2A). Transwell assay was performed to assess the role of IFFO1 in lung cancer cell migration. The result demonstrated that IFFO1 depletion increased migration in NCI-H1299 cells (Fig. 2A, Supplementary Fig. 2B). IFFO1 over-expression in wild-type cells inhibited cell migration in both cell lines (Fig. 2B). Additionally, *IFFO1*^−/−^ cells showed enhanced migration as compared to wild-type NCI-H1299 cells, which can be rescued after re-expression of IFFO1 (Fig. 2C). *IFFO1*^−/−^ cells showed a time-dependent increase in epithelial-mesenchymal transition (EMT) markers N-cadherin and Vimentin, as well as small GTPases Cdc42 mRNA levels (Fig. 2D, Supplementary Fig. 2C), while the E-cadherin level in these cell lines was below the detectable range. In *IFFO1*^−/−^ cells, the expression levels of N-cadherin and Cdc42 exhibit a more pronounced increase over time in comparison to wild-type cells (Fig. 2D–F). Live-cell time-lapse imaging records cell movement over a specific time period, and cell motion trajectories are plotted using the ImageJ plug-in TrackMate [23]. The average speed and total distance of *IFFO1*^−/−^ cells during the recorded 175 min were higher than those of wild-type cells, with the absence of Lamin A as a positive control [23] (Fig. 2G, H, Supplementary Fig. 2D, E). To evaluate the influence of IFFO1 on the metastatic capabilities of lung cancer cells, cells were injected into nude mice through the tail vein. At 8-10 weeks post-injection, the *IFFO1*^−/−^ cohort exhibited a modest increase in focal dark red hemorrhagic regions and surface lesions within the pulmonary tissue. Histopathological examination of the most extensive region of the lung demonstrated a heightened vascular invasion infiltrating the vascular endothelium in the *IFFO1*^−/−^ cohort, as well as an augmented presence of small tumor clusters, forming micro-metastatic foci (Fig. 2I, Supplementary Fig. 2F). Thus, the low expression of IFFO1 in lung cancer promoted cell migration and movement, which might be due to an enhancement of N-cadherin, Vimentin, and Cdc42 expression levels.

### IFFO1 depletion changes GTPase mediated signal transduction

**Fig. 3:**
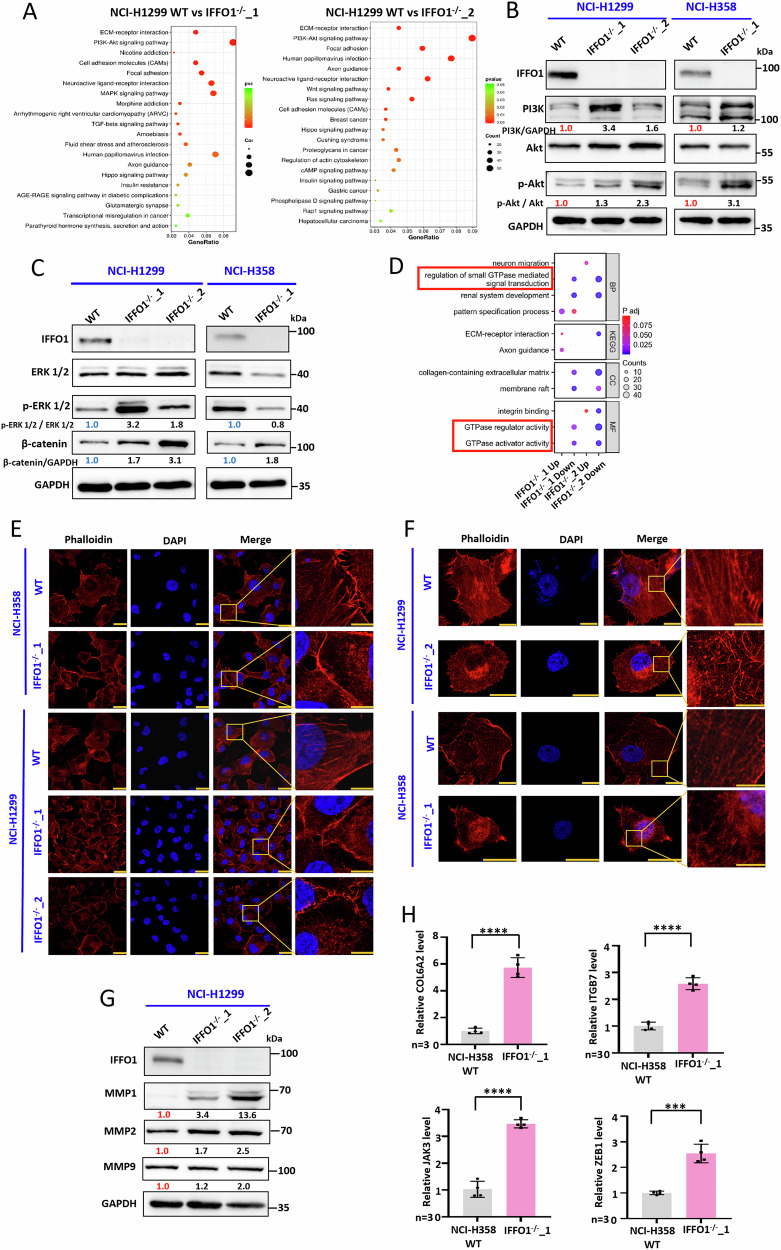
IFFO1 depletion changes GTPase mediated signal transduction and remodels the cytoskeleton. **A** KEGG pathway analysis in two *IFFO1*^−/−^ clones of NCI-H1299 cells. **B** Western blotting of the PI3K/Akt signaling pathway. The relative value of protein expression was obtained by comparing with GAPDH and normalizing with WT cells. **C** Western blotting to determine ERK and β-catenin signal. The relative value of protein expression was obtained by comparing with ERK 1/2 and GAPDH, respectively, and normalizing with WT cells. **D** The comprehensive KEGG analysis of the two *IFFO1*^−/−^ clones of NCI-H1299 cells. TRITC Phalloidin staining of the F-actin cytoskeleton with the Leica TCS SP8 confocal microscope (**E**) and STED super-resolution microscope (**F**). Scale bar = 30 μm. **G** Western blotting for the detection of MMPs. The relative value of protein expression was obtained by comparing with GAPDH and normalizing with WT cells. **H** RT-PCR analysis of mRNA level for COL6A2, ITGB7, JAK3, and ZEB1 in NCI-H358 cells. ***p < 0.001. ****p < 0.0001.

Considering the significance of IFFO1 in the advancement of lung cancer, a transcriptomic analysis was conducted to explore the underlying mechanisms. KEGG pathway analysis showed that *IFFO1*^*-/-*^ cells exhibited remarkable variations in PI3K-Akt signaling pathway, ECM receptor interaction, cell adhesion molecules (CAMs), and focal adhesion (Fig. 3A). *IFFO1*^−/−^ cells exhibited a significant increase in PI3K and phosphorylated Akt expression, indicating the activation of the PI3K-Akt signaling pathway (Fig. 3B). Activated extracellular signal-regulated kinase 1/2 (ERK 1/2) plays a crucial role in inducing cell proliferation [24]. The *IFFO1*^−/−^ NCI-H1299 cells exhibited enhancement in ERK signaling, while the IFFO1-induced proliferation of NCI-H358 cells probably did not depend on ERK (Fig. 3C). As a key signal transducer of the typical WNT signaling pathway, the β-catenin-associated network at the nuclear is closely related to WNT pathway reactivity [25]. *IFFO1*^−/−^ cells demonstrate elevated levels of β-catenin (Fig. 3C). NCI-H1299 cells with depleted IFFO1 exhibited increased accumulation of β-catenin in the nucleus, whereas the overexpression of IFFO1 resulted in a diminished nuclear presence of β-catenin (Supplementary Fig. 2G). A secondary analysis of representative genes within signaling pathways that exert considerable influence revealed that IFFO1 depletion significantly altered the regulation of small GTPase mediated signal transduction, GTPase regulator activity, and GTPase activator activity (Fig. 3D, Supplementary Fig. 3A). Therefore, the depletion of IFFO1 may be significantly associated with the modulation of GTPases.

### IFFO1 depletion remodels the cytoskeleton

Both confocal and stimulated emission depletion (STED) imaging showed the sparse and discontinuous cytoskeleton in IFFO1-depleted cells, accompanied by a weakening of actin bundles (Fig. 3E, F). In addition, *IFFO1*^−/−^ NCI-H1299 cells displayed increased levels of the matrix metalloproteinases (MMPs) proteins MMP1, MMP2, and MMP9 (Fig. 3G). In the present study, NCI-H358 cells were used for further validation to reduce the sequencing bias. The mRNA levels of Collagen VI α2 (COL6A2), the EMT-associated transcription factor ZEB1, the Janus kinase (JAK) family member JAK3, and the oncogene *C-MAF* target ITGB7 in NCI-H358 *IFFO1*^−/−^ cells were significantly increased (Fig. 3H), and this finding was consistent with our RNA-seq results in NCI-H1299 cells (Fig. 3A). These results indicated that the cytoskeleton structure was altered following IFFO1 depletion.

### IFFO1 interacted with the Rho-GTPase IQGAP3 through the 2 A coiled-coil domain

**Fig. 4:**
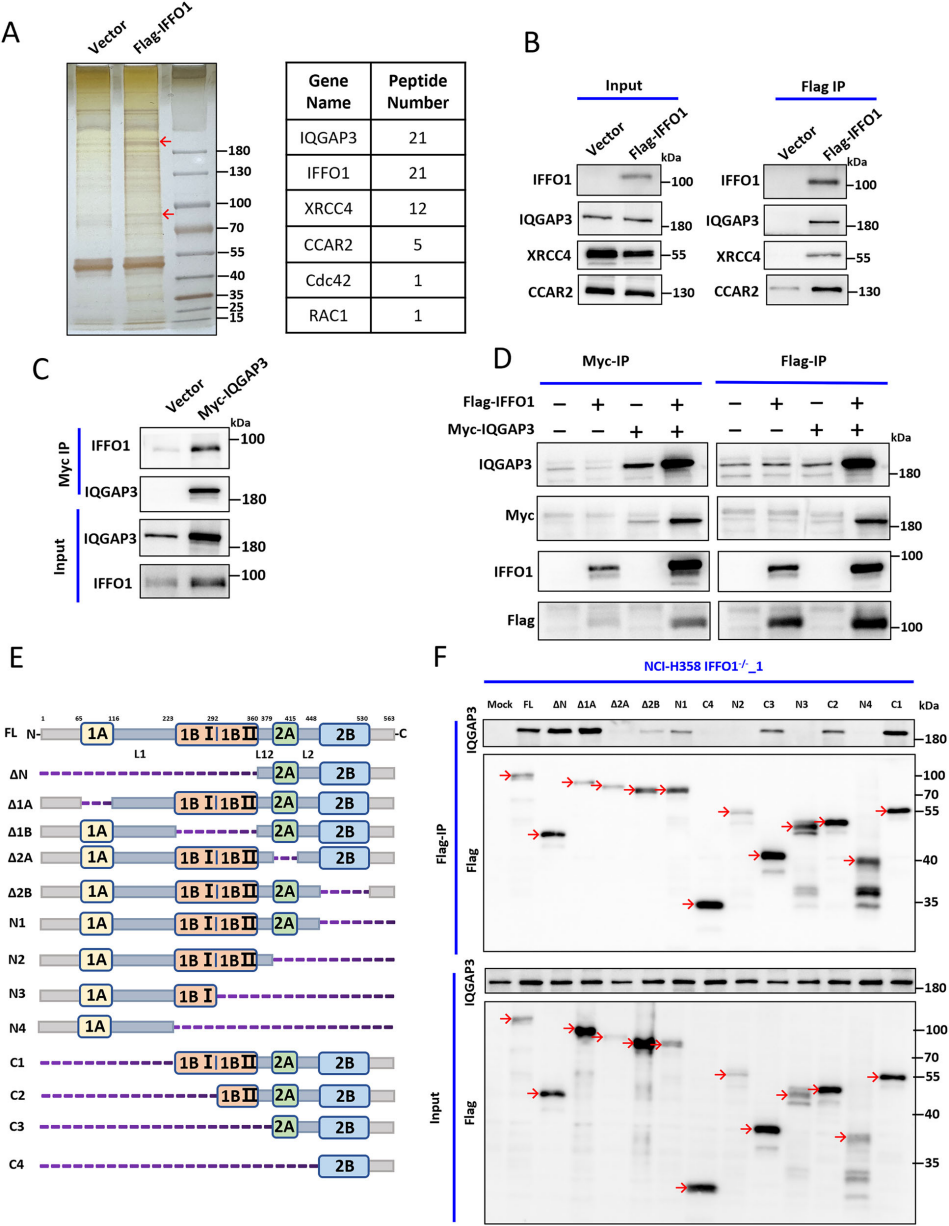
IFFO1 interacted with the Rho-GTPase protein IQGAP3 through the 2 A coiled-coil domain. **A** Silver staining and peptide identification after Flag-IFFO1 immunoprecipitation (IP). **B** Western blotting of proteins interacting with IFFO1. **C** IP results for Myc-tagged IQGAP3. **D** The co-immunoprecipitation (co-IP) results of Flag-tagged IFFO1 and Myc-tagged IQGAP3 in *IFFO1*^−/−^ cells. **E** Structure of IFFO1 and different truncated mutants. FL, full-length. **F** The IP results of different truncated mutants of IFFO1.

To conduct the protein network of IFFO1 interactions in tumor cells, co-immunoprecipitation (co-IP) and mass spectrometry (IP-MS) analysis were performed using Flag-tagged IFFO1. The IP-MS revealed an interaction between the scaffold protein IQGAP3 and IFFO1 (Fig. 4A). IQGAP3 is typically located between the cellular junctions of epithelial cells and is closely associated with cytoskeleton, cell proliferation, cell adhesion, vascular invasion, the invasion and metastasis of cancer cells, and the regulation of cell motility [15, 16, 26]. The IP results confirmed the interaction between IFFO1 and IQGAP3, while IFFO1 did not affect IQGAP3 expression (Fig. 4B, Supplementary Fig. 3B). Myc-tagged IQGAP3 were overexpressed, and the results showed that IFFO1 and IQGAP3 exhibited a bidirectional interaction (Fig. 4C). The co-IP in *IFFO1*^−/−^ NCI-H1299 cells following expression of Flag-IFFO1 and Myc-IQGAP3 showed the consistent results (Fig. 4D). This finding indicates a direct interaction between IFFO1 and IQGAP3. The structure of IFFO1 showed the presence of four coiled-coil domains, namely 1 A, 1B, 2 A, and 2B, and these domains were linked by linkers (L1, L12 and L2) (Fig. 4E). The truncated mutants of each domain were expressed in *IFFO1*^−/−^ cells to study the characteristics and significance of the interaction between IFFO1 and IQGAP3 (Fig. 4E). The IP results showed that IFFO1 lost its interaction with IQGAP3 only after deletion of the 2 A coiled-coil domain (Fig. 4F, Supplementary Fig. 4A–C). Then we performed a more focused truncation analysis within the 2 A coiled-coil domain to elucidate the specifics of the interaction between IFFO1 and IQGAP3. The IP results showed that these truncated mutants exhibited a marginally reduced interaction with IQGAP3 compared to the full-length IFFO1, yet demonstrated a stronger interaction than the mutants that lacked the 2 A coiled-coil domain (Supplementary Fig. 4D). This finding implies that the structural integrity of the 2 A coiled-coil domain is essential for the interaction between IFFO1 and IQGAP3. We constructed the truncated mutants according to the functional domains of IQGAP3 (Supplementary Fig. 5). Both IP and GST pull-down assays suggested that several domains within IQGAP3 exhibit interactions with IFFO1 (Supplementary Fig. 5A, B). Thus, IFFO1 interacted with IQGAP3 through the 2 A coiled-coil domain.

### The migration of IFFO1-deficient cells is mediated by IQGAP3

**Fig. 5:**
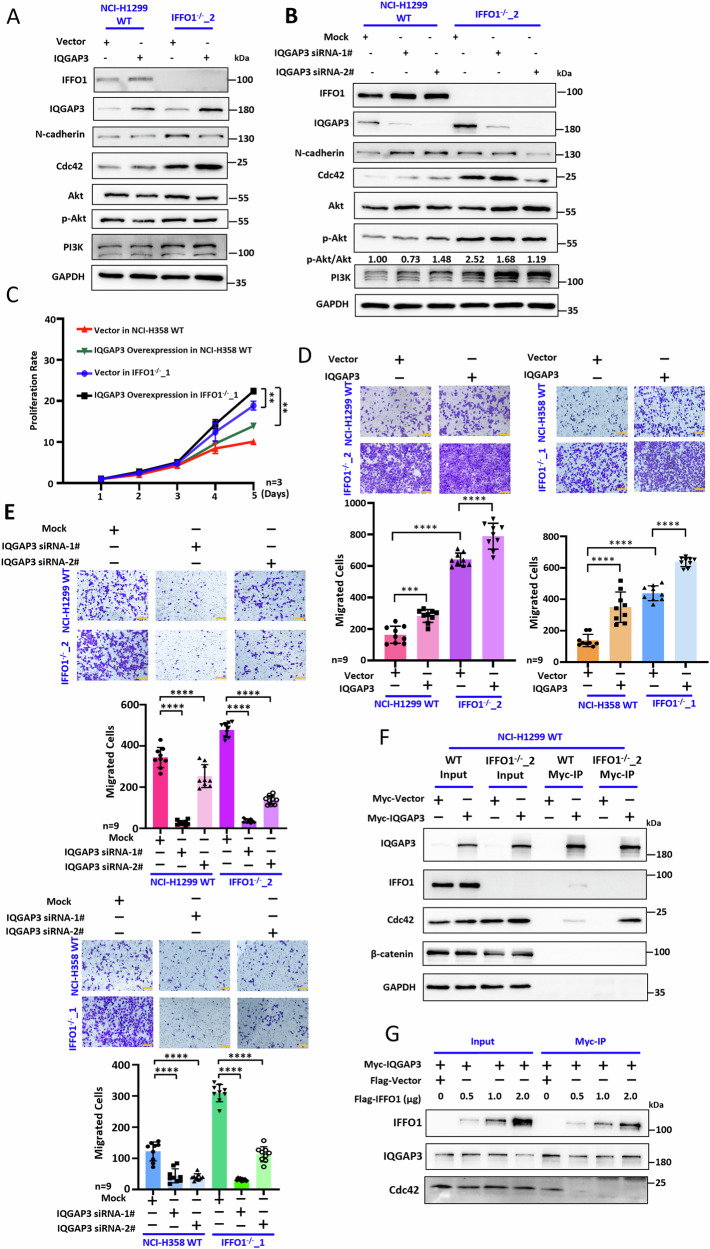
The migration of IFFO1-deficient cells is mediated by IQGAP3-Cdc42. Western blotting detection after IQGAP3 expression (**A**) and IQGAP3 depletion (**B**) in WT and *IFFO1*^−/−^ NCI-H1299 cells. The relative value of protein expression was obtained by comparing with Akt and normalizing with Mock cells. **C** Cell proliferation after IQGAP3 expression. Transwell detection and statistics after IQGAP3 expression (**D**) and IQGAP3 depletion (**E**) in WT and *IFFO1*^−/−^ cells. **F** IP results for Myc-tagged IQGAP3 in WT and *IFFO1*^−/−^ NCI-H1299 cells. **G** IP results for Myc-tagged IQGAP3 followed by the expression of Flag-IFFO1. **p < 0.01. ***p < 0.001. ****p < 0.0001.

We conducted a comprehensive assessment of the function of IQGAP3 in lung cancer cells. The overexpression of IQGAP3 results in elevated levels of Cdc42 in NCI-H1299 cells, and this effect exhibit further enhancement in cells lacking IFFO1 (Fig. 5A). Furthermore, the silencing of IQGAP3 leads to reduced N-cadherin and Cdc42 in *IFFO1*^−/−^ cells (Fig. 5B). Compared to the expression in wild-type cells, the over-expression of IQGAP3 in *IFFO1*^*-/-*^ cells further promotes cell proliferation (Fig. 5C). Additionally, the over-expression of IQGAP3 accelerates cell migration, while the knockdown of IQGAP3 significantly impairs migration in both wild-type and *IFFO1*^−/−^ cells (Fig. 5D, E). To eliminate the effect of cell proliferation on cell migration, we subjected the cells to a 2-hour pre-treatment with 2 µg/ml Mitomycin C (MMC) prior to the execution of the Transwell assay [27]. The findings corroborated those presented in Fig. 5E, indicating that the depletion of IQGAP3 in *IFFO1*^−/−^ cells led to a decrease in the number of migrating cells (Supplementary Fig. 6A). Consequently, it can be inferred that the migration of IFFO1-deficient cells is, at least in part, mediated by IQGAP3.

### IFFO1 and IQGAP3 interaction inhibits the binding of IQGAP3 to the effector Cdc42

IQGAP3 is known to interact with the active form of Ras (GTP-bound), as well as with Cdc42 and Rac1 [15]. To elucidate the specific molecular mechanism underlying the interaction between IFFO1 and IQGAP3, we investigated the impact of IFFO1 on active Ras. Our findings indicated that IFFO1 did not exert a significant effect on active Ras in two distinct lung cancer cell lines (Supplementary Fig. 6B). Therefore, IFFO1 may modulate the crosstalk between IQGAP3 and other effectors. Notably, Cdc42 was found to be significantly elevated in *IFFO1*^−/−^ cells (Fig. 2D), whereas Cdc42 was not detected in the immunoprecipitants of IFFO1 (Fig. 4A, B). Furthermore, the interaction between Cdc42 and IQGAP3 was markedly enhanced in *IFFO1*^−/−^ cells (Fig. 5F). Importantly, upon re-expression of IFFO1 in *IFFO1*^−/−^ cells, the binding of Cdc42 to IQGAP3 was observed to decrease in a manner that was dependent on the amount of IFFO1 (Fig. 5G). Thus, it can be concluded that IFFO1 inhibits IQGAP3 function by interfering with its interaction with Cdc42.

### IFFO1 and IQGAP3 interaction is responsible for cell migration and mobility

**Fig. 6:**
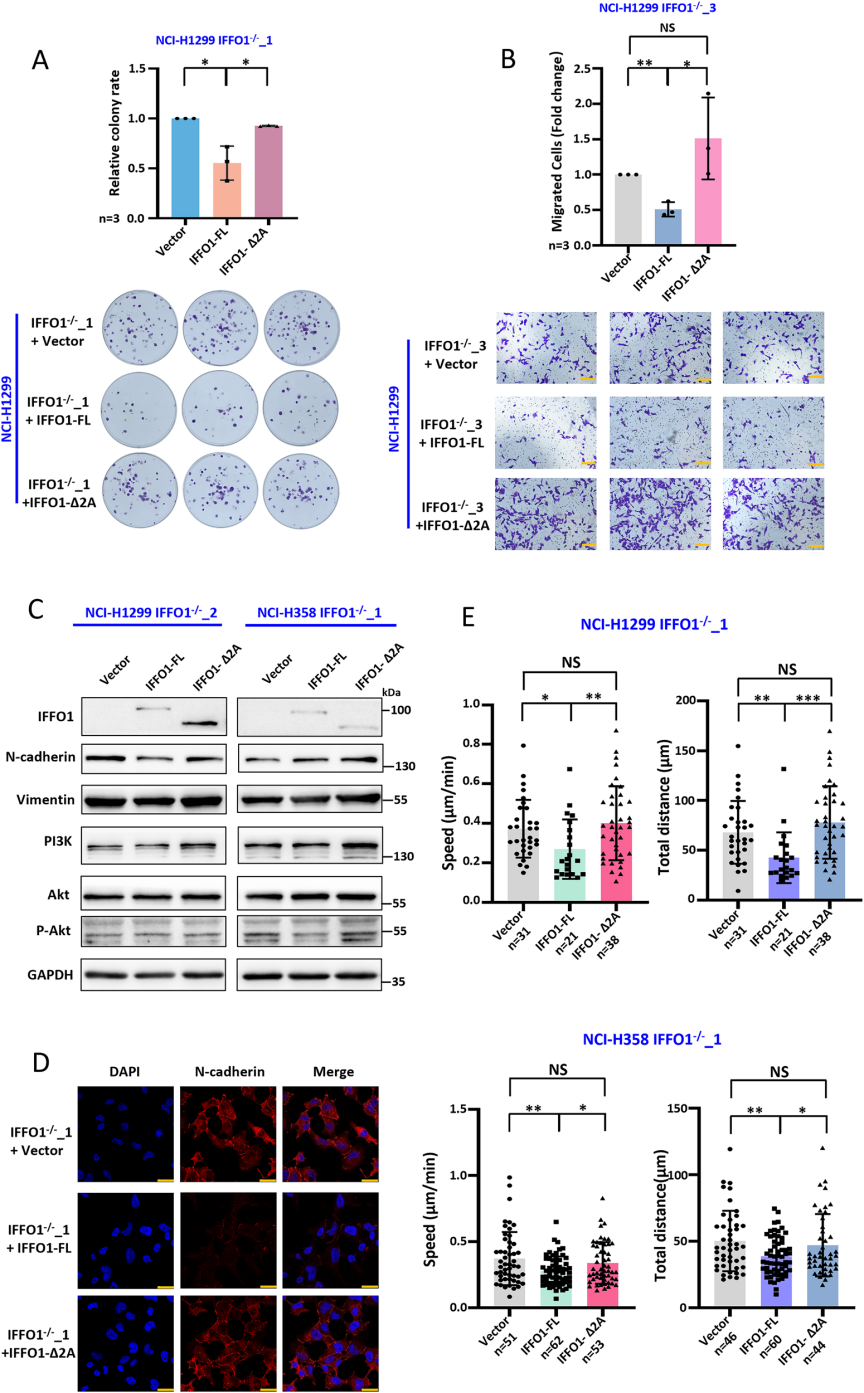
IFFO1 and IQGAP3 interaction is responsible for cell migration and mobility. Clone formation **A**, the migration **B**, Western blotting of N-cadherin, Vimentin, and PI3K/Akt signaling pathway **C**, immunofluorescence staining of N-cadherin **D**, and speed and total distance during the recorded 175 min **E** after re-expression of full-length IFFO1 and the mutant losing IQGAP3 interaction (∆2 A) in *IFFO1*^−/−^ cells. The scale bars are 100 μm (**B**) and 30 μm (**D**), respectively. *p < 0.05. **p < 0.01. ***p < 0.001. NS, not significant.

We compared the re-expression of full-length IFFO1 and the mutant with loss of IQGAP3 interaction to better confirm the effects of IFFO1 on cell migration and movement. Following IFFO1 depletion, the re-expression of the full-length IFFO1, instead of the 2 A coiled-coil domain deletion mutant, significantly inhibited clone formation (Fig. 6A, Supplementary Fig. 6C) and migration (Fig. 6B). Similarly, following the treatment with MMC, the full-length IFFO1 continues to inhibit the migration of *IFFO1*^−/−^ cells, whereas the 2 A coiled-coil domain deletion mutant exhibits no impact on migration (Supplementary Fig. 6D). The interaction between IFFO1 and IQGAP3 does not influence the active Ras levels within the cells (Supplementary Fig. 6E). Moreover, the N-cadherin, PI3K, and p-Akt levels were significantly reduced after re-expression of full-length IFFO1 in *IFFO1*^−/−^ cells, while there was no significant change following the 2A-deletion mutant re-expression (Fig. 6C, D). Compared to the 2A-deletion mutant, *IFFO1*^−/−^ cells with re-expression of full-length IFFO1 slowed reduced speed and total distance traveled (Fig. 6E, Supplementary Fig. 6F). These results suggested that IFFO1 affects the migration and mobility of lung cancer cells through the interaction with IQGAP3.

### IFFO1 was downregulated in lung cancer and associated with tumor progression

**Fig. 7:**
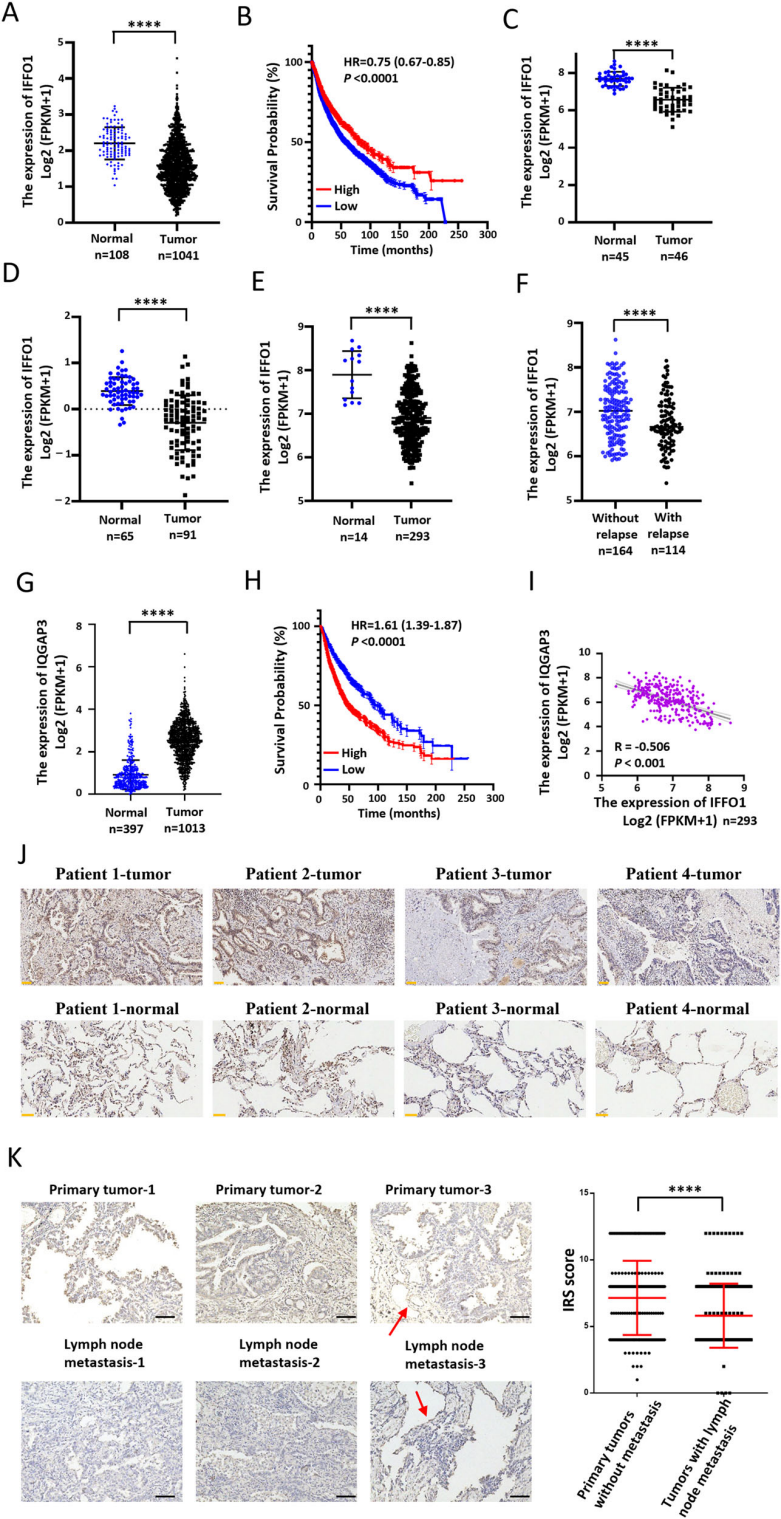
IFFO1 was downregulated in lung cancer and associated with tumor progression. **A** IFFO1 expression between tumor tissues and normal tissues in the TCGA lung cancer dataset. **B** Kaplan–Meier survival analysis of overall survival. Patients were assigned to the high and low expression groups according to the IFFO1 expression level. **C** Microarray analysis of IFFO1 expression between tumor tissues and normal tissues from the GSE18842 dataset. **D** IFFO1 expression profile between tumor tissues and adjacent normal tissues from the GSE19188 dataset. **E** Comparison of IFFO1 expression between tumor samples and non-tumor lung samples from the GSE30219 dataset. **F** Comparison of IFFO1 expression between tumor samples with or without relapse in the GSE30219 dataset. **G** IQGAP3 expression between tumor tissues and normal tissues in the TCGA lung cancer dataset. **H** Kaplan–Meier survival analysis of overall survival according to the IQGAP3 expression level. **I** Correlation analysis of expression levels between IFFO1 and IQGAP3 from the GSE30219 dataset. **J** Immunohistochemical staining of IFFO1 in tumor tissues and adjacent normal tissues from tissue microarray chips (Shanghai Outdo Biotech Company). Scale bar = 50 μm. **K** Immunohistochemical staining and IRS (immunoreactivity score) of IFFO1 in primary tumors without metastasis and tumors with lymph node metastasis. Each group comprises ten tissue sections, from which 20 to 30 regions are randomly selected from each section. These regions are independently evaluated by two pathologists. The red arrow indicates the positive expression of IFFO1 in lung epithelial cells. Scale bar = 50 μm. ****p < 0.0001.

**Fig. 8:**
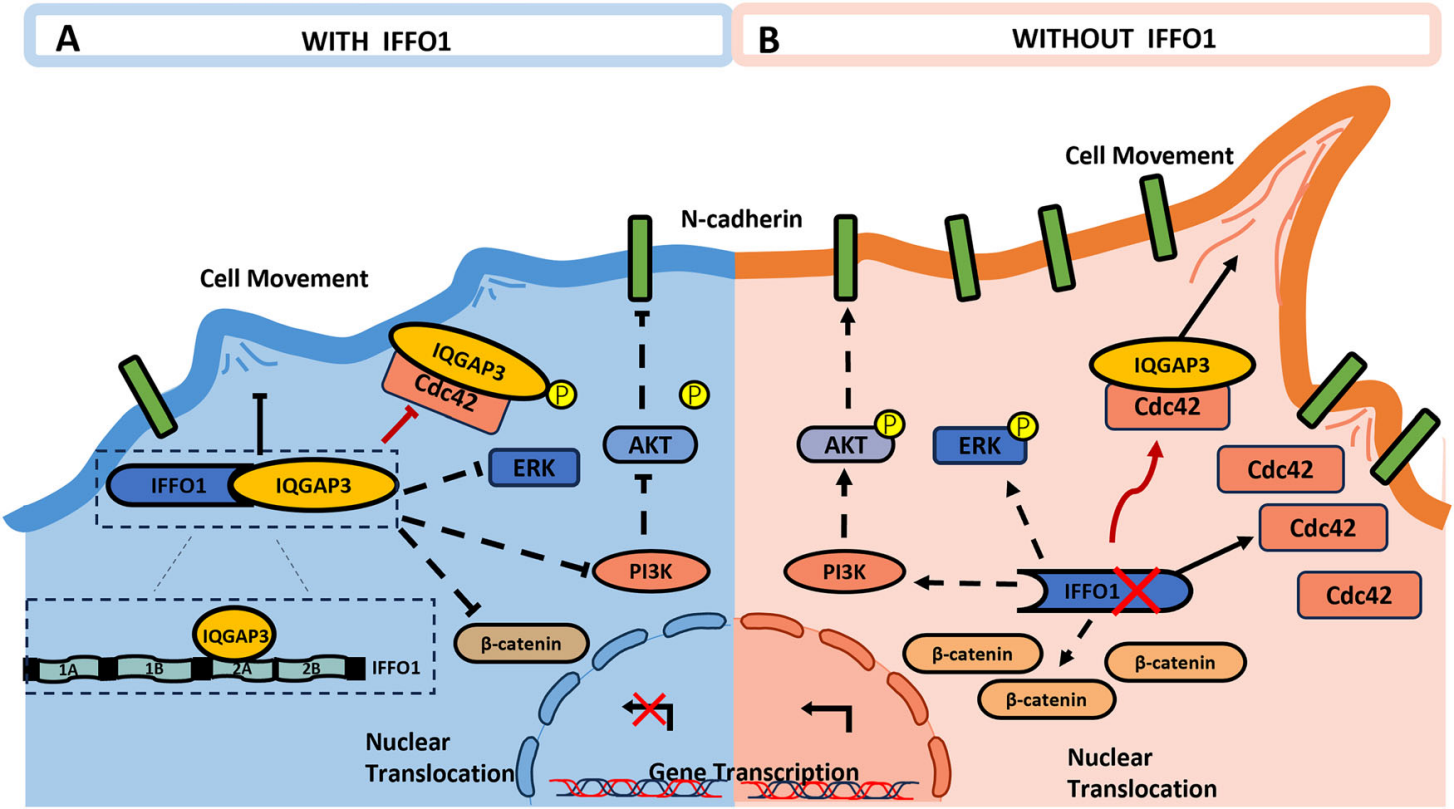
The underlying mechanism of IFFO1 in lung cancer migration. IFFO1 interacts with IQGAP3 through the 2 A coiled-coil domain and inhibits IQGAP3/Cdc42-mediated cell motility and ERK/Akt-mediated cell proliferation. Downregulation of IFFO1 in lung cancer cells promotes N-cadherin expression and cell motility through the release of IQGAP3.

We then determined the expression level of *IFFO1* to understand its physiological role in lung cancer patients. The RNA sequencing (RNA-seq) data from 1041 lung cancer tissues and 108 normal adjacent tissues showed lower IFFO1 expression levels in tumors than in normal tissues (Fig. 7A). The Kaplan–Meier survival curve indicated a positive correlation between IFFO1 expression and the overall survival in lung cancer patients (Fig. 7B). The IFFO1 expression level was lower in the two subtypes of lung cancer (lung adenocarcinoma and lung squamous cell carcinoma) than in normal tissues (Supplementary Fig. 7A, B). The microarray analysis of the GSE18842, GSE19188, and GSE30219 datasets showed downregulation of IFFO1 expression in tumor tissues (Fig. 7C–E), and the IFFO1 expression level decreased with the increasing clinical staging (Supplementary Fig. 7C, D). More importantly, IFFO1 expression in the tumor tissues of patients with relapse was significantly lower than that in patients without relapse in the GSE30219 dataset (Fig. 7F). IQGAP3 expression in lung cancer tissues is markedly elevated compared to adjacent non-cancerous tissues, as evidenced by data from both the TCGA database and the above GEO databases (Fig. 7G, Supplementary Fig. 7E–G). Furthermore, patients with relapse exhibit higher IQGAP3 expression levels than those without relapse (Supplementary Fig. 7H). Notably, patients categorized within the high IQGAP3 expression group demonstrate reduced overall survival rates (Fig. 7H). A significant negative correlation was observed between the expression levels of IFFO1 and IQGAP3 in the GSE30219 database (Fig. 7I). The IFFO1 staining in tissue microarray indicated that the alveolar epithelial cells adjacent to the tumor demonstrate elevated levels of IFFO1 expression in comparison to that in tumor tissues (Fig. 7J). A retrospective analysis was performed on a cohort of 20 lung cancer patient specimens, comprising 10 cases of non-metastatic primary tumors and 10 cases of tumors exhibiting varying degrees of lymph node metastasis. Immunohistochemical analysis of IFFO1 was conducted on the tissue sections. In alignment with the findings presented in Fig. 7J, the expression of IFFO1 was notably elevated in alveolar epithelial cells, stromal cells, and infiltrating immune cells, as highlighted by the red arrows in Fig. 7K. The IRS score revealed that IFFO1 expression in tissues exhibiting lymph node metastasis was significantly lower compared to that in primary tumor tissues (Fig. 7K). These findings suggested that IFFO1 was associated with the malignant progression of lung cancer patients by regulating the interaction between IQGAP3 and effector proteins (Fig. 8).

## Discussion

Tumor migration requires the synchronous coordination of microtubule polymerization/depolymerization and focal adhesion (FA) assembly/disassembly, as well as changes in global cell polarity regulated by Rho GTPase [28]. Microtubule-targeting agents have been widely tested and used in clinical applications either individually or in combination [29]. Actin polymerization/depolymerization are involved in metastasis, ECM degradation, perception and transmission of environmental mechanical forces, intracellular signal transduction, and material transport [30]. IFs distributed in the cytoplasm and nucleus are involved in physiological and pathological processes, including cellular, mechanical, and morphological support [31]; subcellular compartmentalization [32]; homeostasis of protrusion related to migration [33]; and signal transduction across the cytoplasm and nucleus [34]. The role of IFs in human tumors has been reported since the 1970s [35]. However, thus far, research on IFs in tumors has focused on phenomenon, and the influence of tumor types and interacting molecules on the related mechanisms of IFs requires further studies.

Different IF proteins have diverse effects on tumor progression. The low expression of Lamin A/C in breast cancer may help compensate nuclear deformation during migration [36], whereas the high expression of Lamin A/C is associated with a better prognosis [37]. Nuclear signals are involved in resisting mechanical changes during cell migration and interfere with biochemical signals of malignant transformation [38]. Our previous study showed that IFFO1 shares a similar structure and function with Lamin A/C in the nucleus [7], and it was more widely distributed in the nucleus and cytoplasm. To develop new tumor targets, it is therefore crucial to study the precise pathological mechanisms through which IFFO1 plays a role in tumor progression, which involves perception of the mechanical force and signal transmission to the nucleoskeleton and cytoskeleton. Compared to the known pharmacological targets targeting actin and microtubules, investigation of the function of IFs distributed in different subcellular regions is critical for developing IF-targeted drugs to treat diseases. Akt-dependent phosphorylation of vimentin at Ser39 promoted EMT and cell migration [39]. Our present research indicated that the PI3K-Akt signaling pathway was activated in IFFO1-deficient cells, which was accompanied by enhanced cell motility. Because of the similarity in structure and function between IFFO1 and Lamin A/C, we speculate that IFFO1 may undergo PTMs during tumor progression. The role of IFs in the TME depends on the functional regulation of PTMs, and the function of IFs in biological activities requires further research.

The metastatic ability of lung cancer can be predicted based on their genomic and transcriptome characteristics [40], and the immunological landscape of the TME is also involved in reshaping the developmental characteristics of the tumor during metastasis [41]. Our research provided evidence that IFFO1 could be considered not only as a scaffold protein, but also as a regulatory factor for intracellular signaling. IFFO1 could control the overall phenotype of cells by regulating its interaction with the crucial GTPase-activating protein IQGAP3 (Fig. 8). In tumors, Cdc42 is generally found to be upregulated rather than mutated, indicating that it may serve as a viable target for therapeutic intervention [42]. One of the oncogenic effectors associated with Cdc42 is PI3K, and the Akt signaling pathway activated by the EGFR-Cdc42 is significantly linked to tumor recurrence and resistance [43, 44]. Consequently, Cdc42 inhibitors present promising chemotherapeutic sensitizers in the treatment of tumors characterized by Ras and EGFR mutations [44]. Our research indicates that the interaction between IQGAP3 (which is released from IFFO1-deficient cells) and Cdc42 enhances the downstream activities. This interaction may contribute to the aberrant activation of the PI3K-Akt signaling pathway observed in IFFO1-deficient cells. Given the challenge of the “undruggable” properties of Rho GTPases and the intricate downstream signaling pathways, current strategies for Cdc42 inhibition primarily focus on obstructing interactions with guanine nucleotide exchange factors (GEFs) or inhibiting nucleotide binding [45]. Our findings suggest that IFFO1 expression decreased the interaction between Cdc42 and its effectors, thereby mitigating Cdc42-related malignant progression. This approach may offer a more moderate regulatory framework for the inhibition of Cdc42 and pave the way for innovative combination therapies.

Presently, most knowledge on IF function has been mainly derived from pathological research in different disease backgrounds. Based on the current understanding of the role of IFs, they cannot be simply regarded as a static scaffold network but are thought to participate in dynamic cell regulation by responding to extracellular signals and instantly remodeling local cellular structures. Nevertheless, several open questions need to be considered in future studies, including (1) how to differentiate between the dominant role of IFs and their accompanying phenomena in tumor progression; (2) are there fluctuations in the expression, distribution transition, and functional redundancy in IFs at different stages of tumor progression; and (3) can IF become a nuclear-cytoplasmic hub that promotes tumor cell signaling and thus be considered a target for cancer treatment? Overall, the present study indicated that IFFO1 regulated the dynamic response of cell mobility through protein-protein interactions and served as a signaling molecule in cells to a certain extent. This suggested an exciting possibility of targeting the more flexible IFs within cells to regulate tumor progression through multiple approaches.

## Supplementary information

Supplementary materials

## Data Availability

The datasets used and/or analyzed during the current study are available from the corresponding author upon reasonable request.
