# Supplementary material for: Intermediate filament IFFO1 negatively regulates the migration of lung cancer cells by inhibiting the IQGAP3-Cdc42 interaction
Source: Cell Death Dis. 2025 Jul 10;16(1):509. doi: 10.1038/s41419-025-07846-z (PMC12241364; doi:10.1038/s41419-025-07846-z)

Supplementary Figure 1

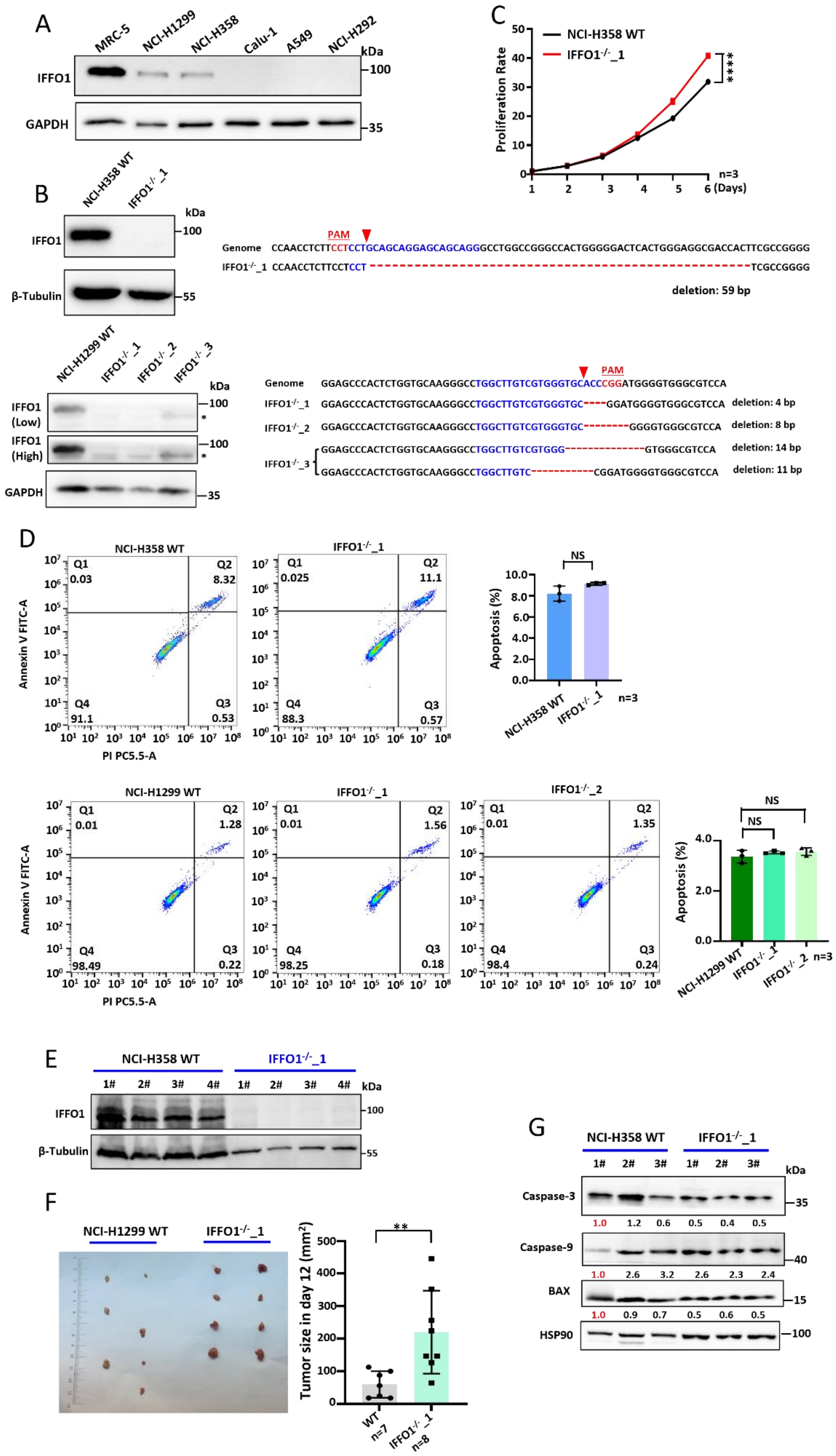

Supplementary Figure 1. IFFO1 depletion increases tumor growth. (A) IFFO1 expression in human embryonic lung fibroblast cell line MRC-5 and lung cancer cell lines. (B) Protein detection and genomic mutation in CRISPR/Cas9 knockout clones from the NCI-H1299 cell line and the NCI-H358 cell line targeting *IFFO1*. *, non-specific band. (C) Cell proliferation in NCI-H358 cells. (D) Apoptosis detection in WT and *IFFO1*$^{-/-}$ cells. (E) IFFO1 expression level in mouse xenografts derived from NCI-H358 cells. (F) Tumor xenografts in BALB/c nude mice with NCI-H1299 cells after 12 days of transplantation. The tumor volume before termination of recording is too small to measure. (G) Caspase-3, caspase-9, and BAX expression levels in mouse xenografts derived from NCI-H358 cells. The relative value of protein expression was obtained by comparing with GAPDH and normalizing with wild-type xenograft (1#). **$p < 0.01$. ****$p < 0.0001$. NS, not significant.

Supplementary Figure 2

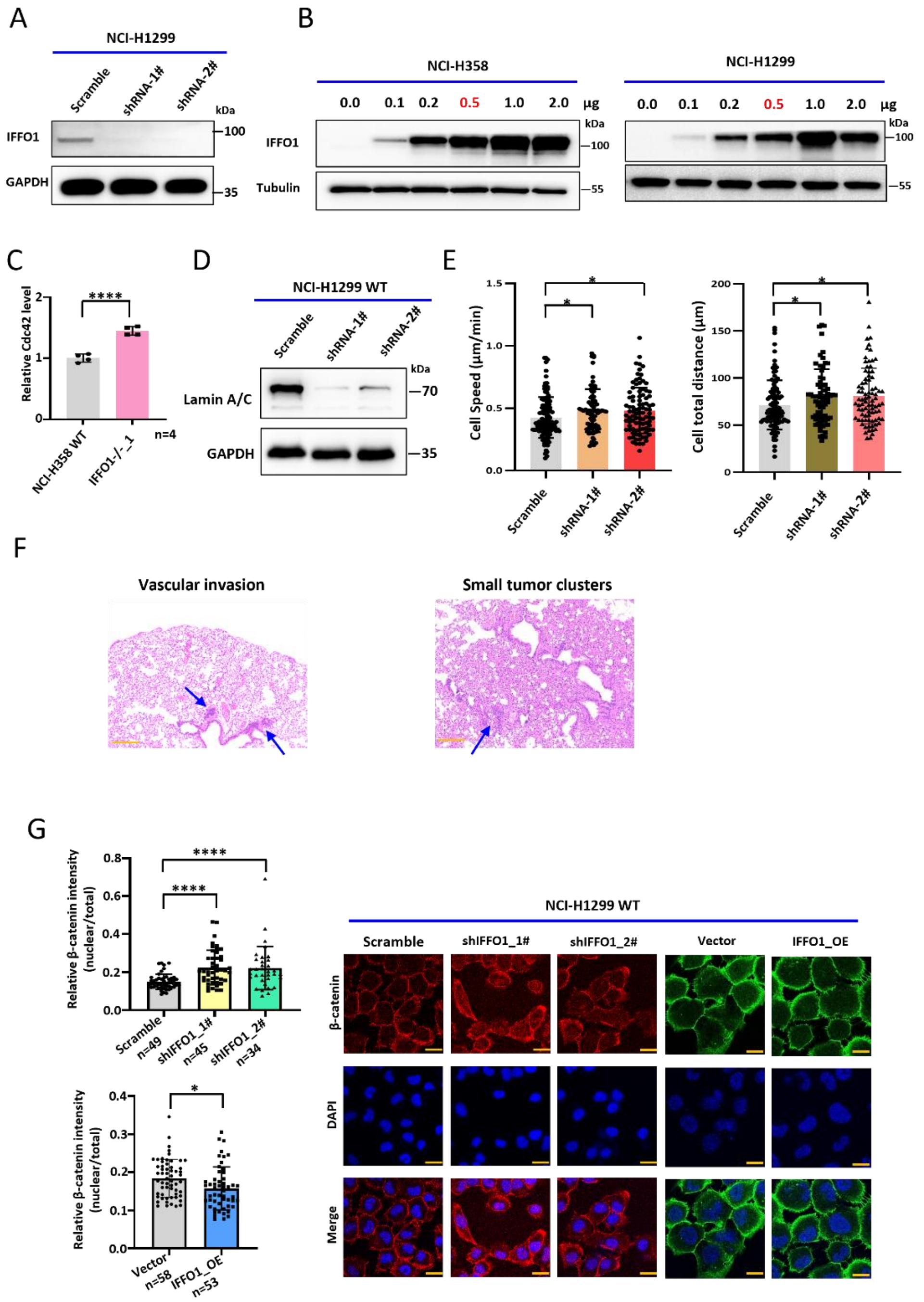

Supplementary Figure 2. IFFO1 depletion increases the migration of lung cancer cells. (A) IFFO1 level following IFFO1 depletion. (B) IFFO1 expression at different transfection levels in NCI-H1299 and NCI-H358 cells. In subsequent cell transfection, a plasmid quantity of 0.5 μg was used. (C) RT-PCR analysis of Cdc42 mRNA level. Protein expression (D) and the average speed/total distance (E) after Lamin A/C depletion in NCI-H1299 cells. (F) Micro-metastatic foci formed by vascular invasion (Left) and small tumor clusters (Right). Scale bar = 50 μm. (G) The immunofluorescence staining (right) and statistical analysis of the nuclear entry ratio of β-catenin (left). OE, over-expression. Scale bar = 30 μm. $*p < 0.05$. $****p < 0.0001$.

Supplementary Figure 3

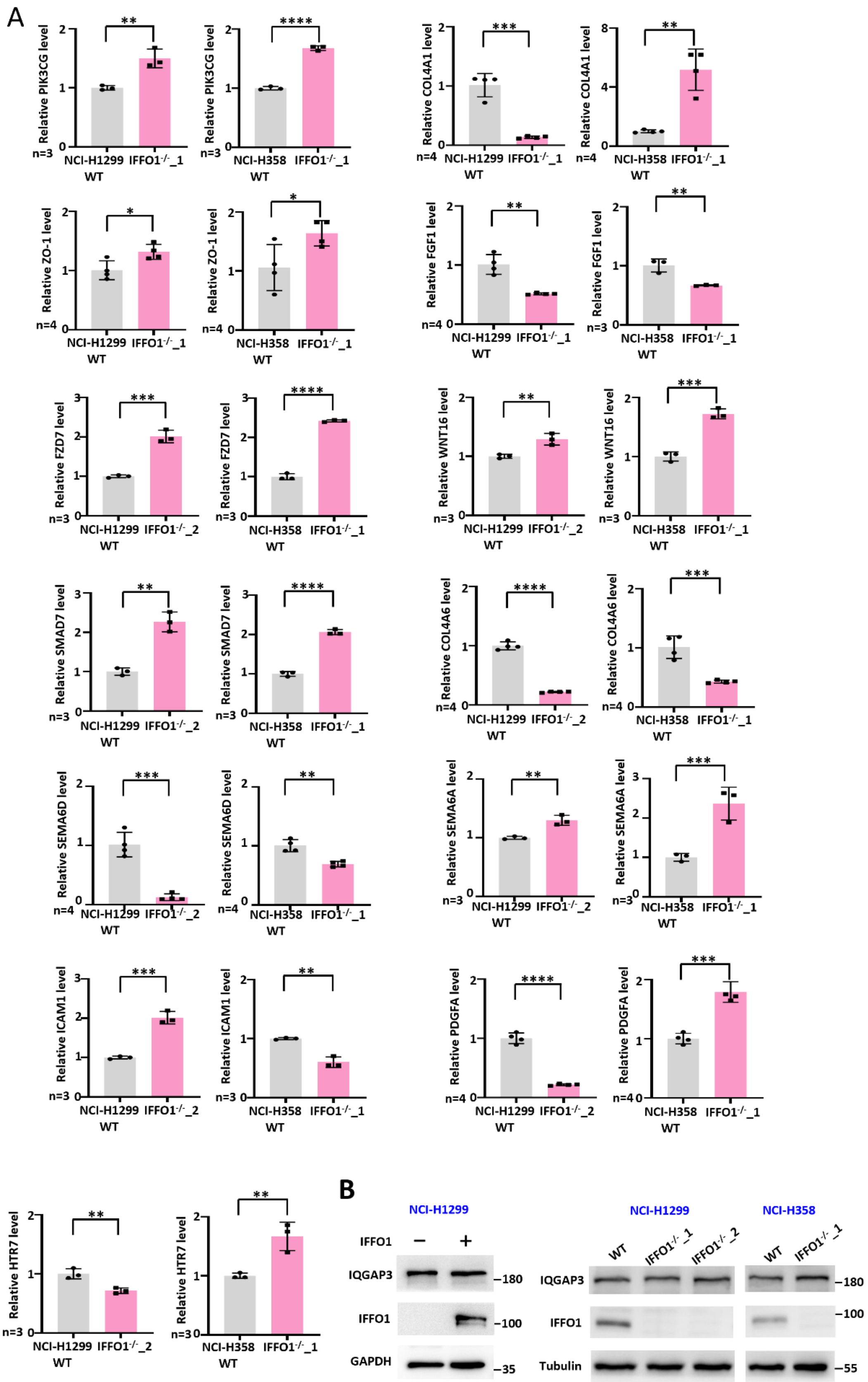

Supplementary Figure 3. IFFO1 depletion changes GTPase mediated signal transduction. (A) Genes expression in PI3K-Akt signaling pathway, MAPK signaling pathway, Focal adhesion, ECM-receptor interaction, Cell adhesion molecules (CAMs), Axon guidance, Ras signaling pathway, Regulation of actin cytoskeleton, Hippo signaling pathway, JAK-STAT signaling pathway, cGMP-PKG signaling pathway and Wnt signaling pathway. (B) IQGAP3 expression after IFFO1 over-expression and depletion. *$p < 0.05$. **$p < 0.01$. ***$p < 0.001$. ****$p < 0.0001$.

Supplementary Figure 4

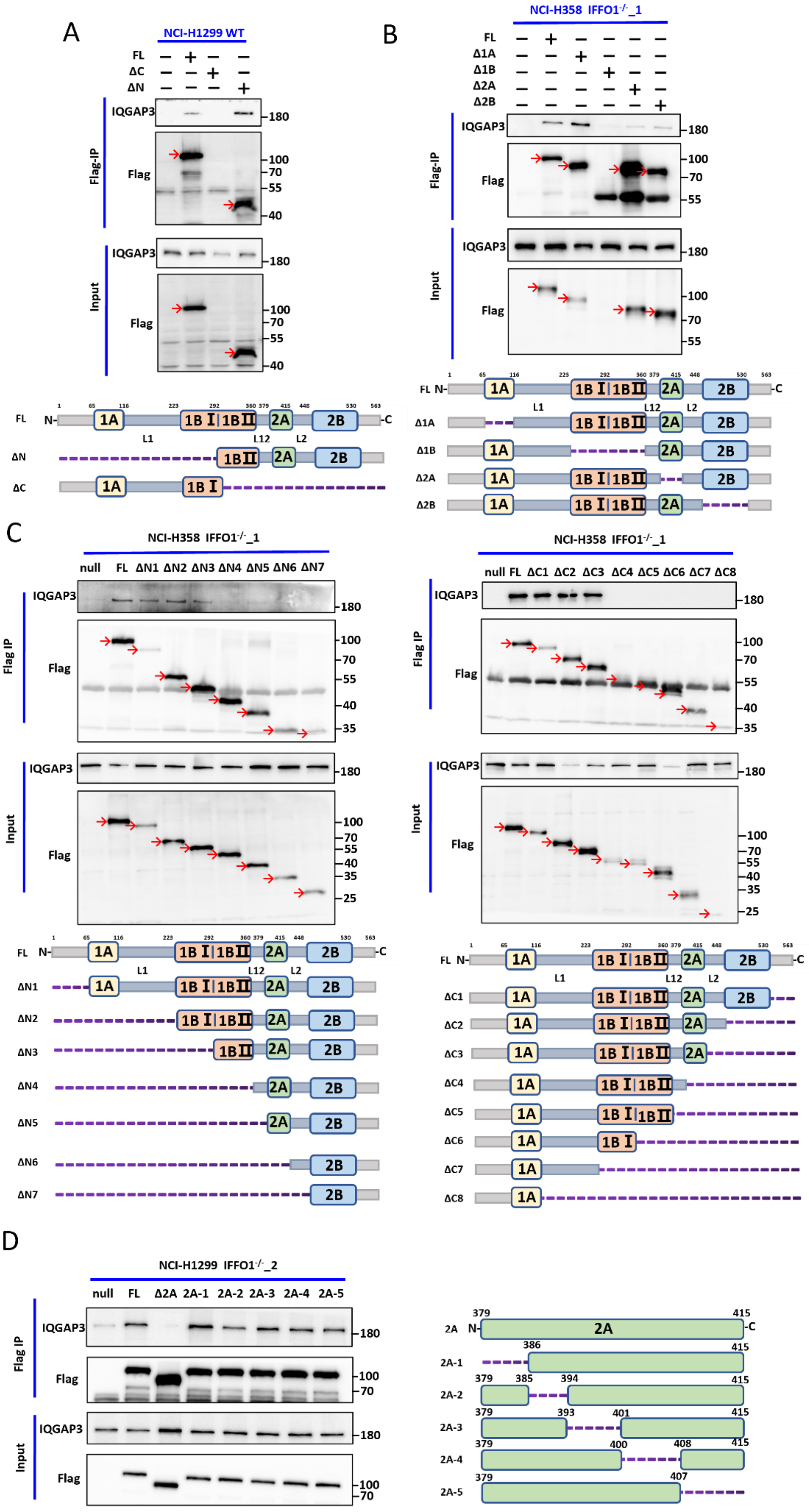

Supplementary Figure 4. IFFO1 interacts with IQGAP3 through the 2A coiled-coil domain. (A) IP results for the interaction of N-terminal and C-terminal mutants of IFFO1 with IQGAP3. The C-terminal mutant was not expressed, while the interaction between the N-terminal mutant and IQGAP3 was not affected. FL, full-length. (B) IP results for the interaction of four main domain deletion mutants (1A, 1B, 2A, and 2B) of IFFO1 with IQGAP3 in *IFFO1*$^{-/-}$ cells. The deletion mutant of the 1B domain was not expressed, while the interaction between the 2A and 2B deletion mutants with IQGAP3 was reduced. (C) The IP results of different truncated mutants of IFFO1 in *IFFO1*$^{-/-}$ cells. The mutants of ΔN2, ΔN3, ΔN5 and ΔN7 are the same mutant as the mutants of C1, C2, C3 and C4 in the main text, respectively. The mutants of ΔC2, ΔC4, ΔC6 and ΔC7 are the same mutant as the mutants of N1, N2, N3 and N4 in the main text, respectively. (D) The IP results of different truncated mutants of 2A coiled-coil domain of IFFO1 in *IFFO1*$^{-/-}$ NCI-H1299 cells.

Supplementary Figure 5

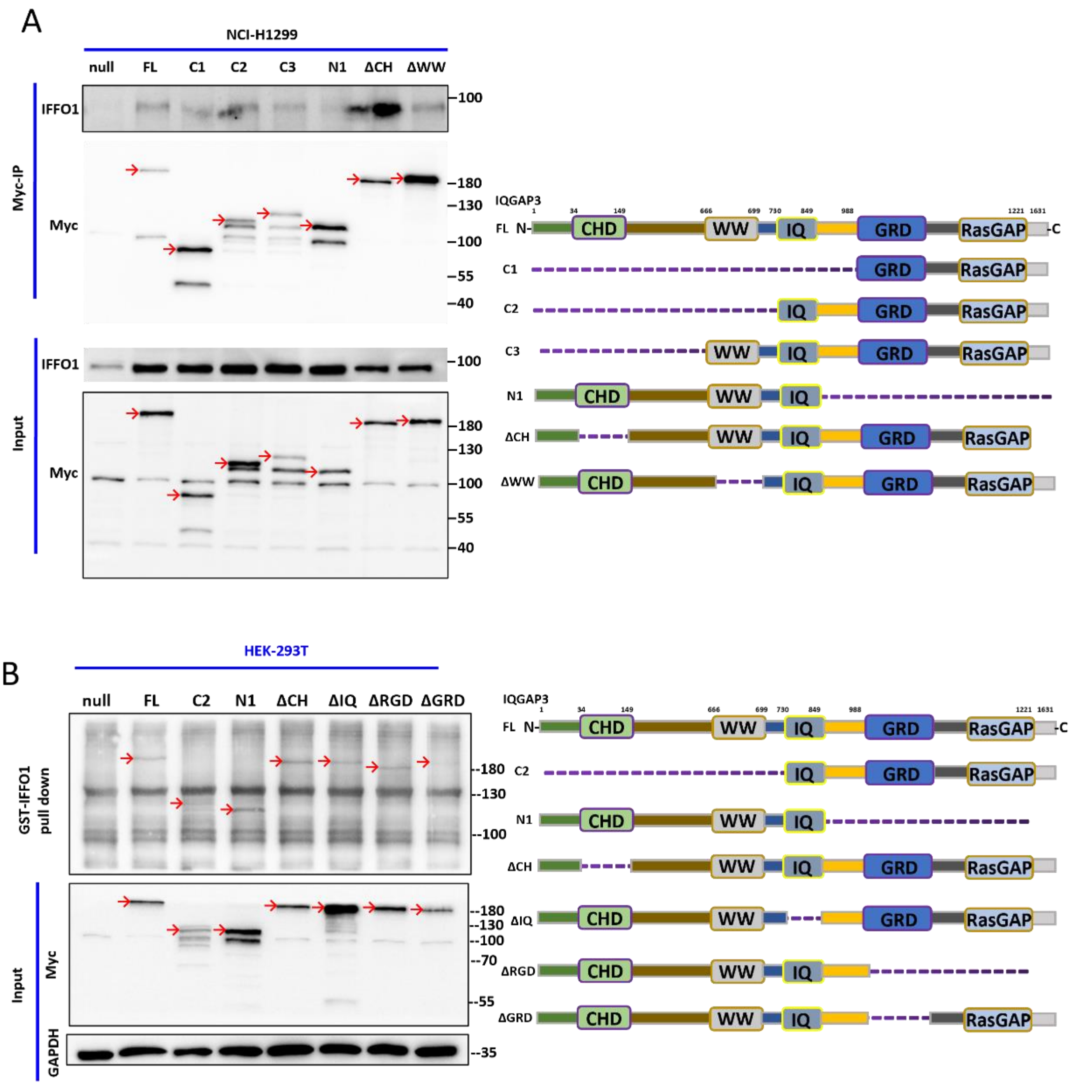

Supplementary Figure 5. IQGAP3 interacts with IFFO1 through multiple domains. (A) IP results for the interaction of IFFO1 in truncated Myc-IQGAP3 mutants in NCI-H1299 cells. FL, full-length. (B) Pull down results between GST-IFFO1 with truncated Myc-IQGAP3 mutants in HEK-293T cells.

Supplementary Figure 6

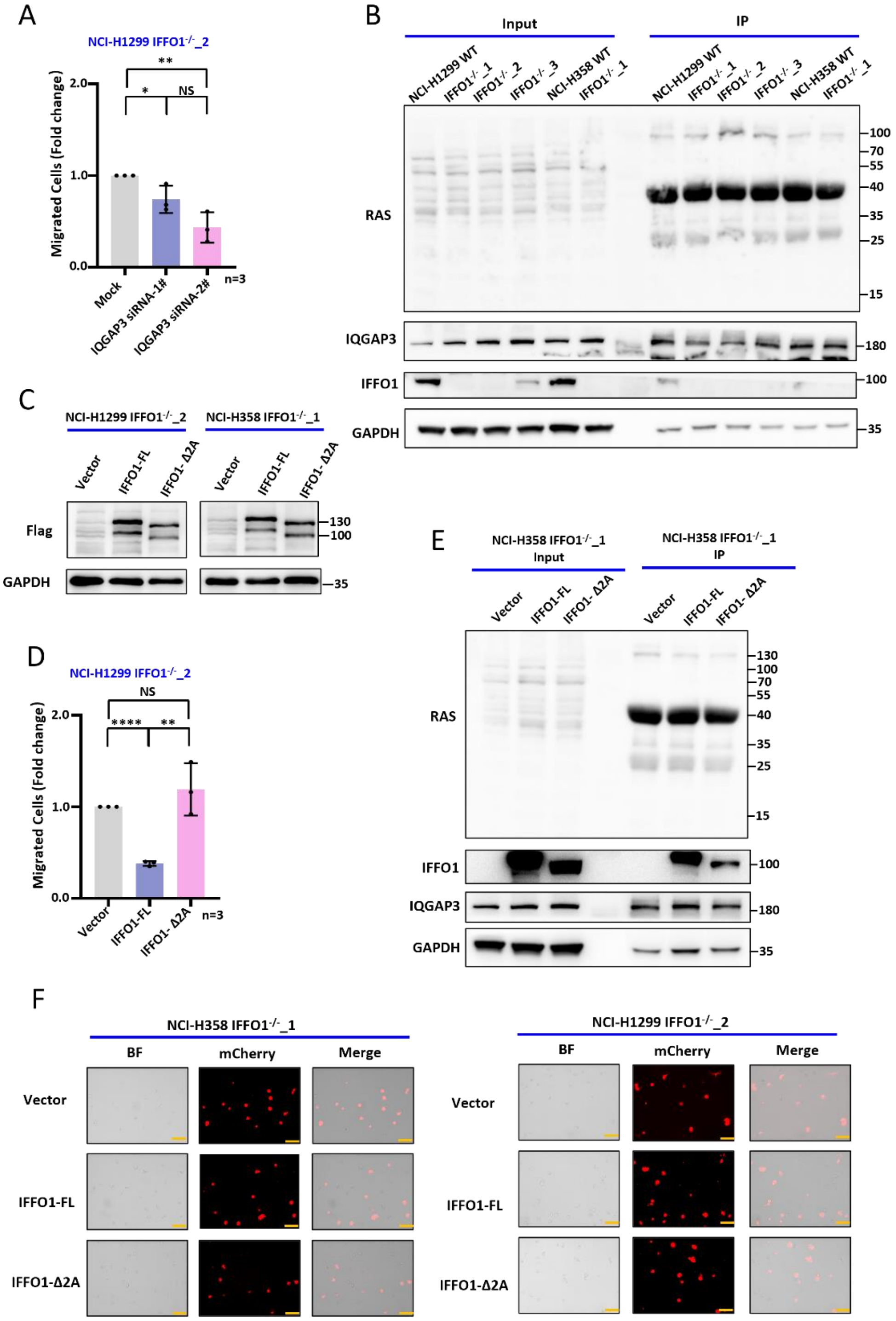

Supplementary Figure 6. IFFO1 and IQGAP3 interaction is responsible for cell mobility but not related to active Ras. (A) Cell migration following the depletion of IQGAP3, subsequent to a 2-hour pre-treatment with 2 µg/ml Mitomycin C (MMC). (B) Active Ras pull-down in WT and *IFFO1*$^{-/-}$ cells. Expression level (C), migration after MMC treatment (D) and active Ras pull-down results (E) after full length and 2A coiled-coil domain deletion of Flag-IFFO1- mCherry expression. (F) Single cells expressing full length and 2A coiled-coil domain deletion of Flag-IFFO1- mCherry were selected for cell motility analysis. Scale bar = 50 µm. *p < 0.05.**p < 0.01. ****p < 0.0001. NS, not significant.

Supplementary Figure 7

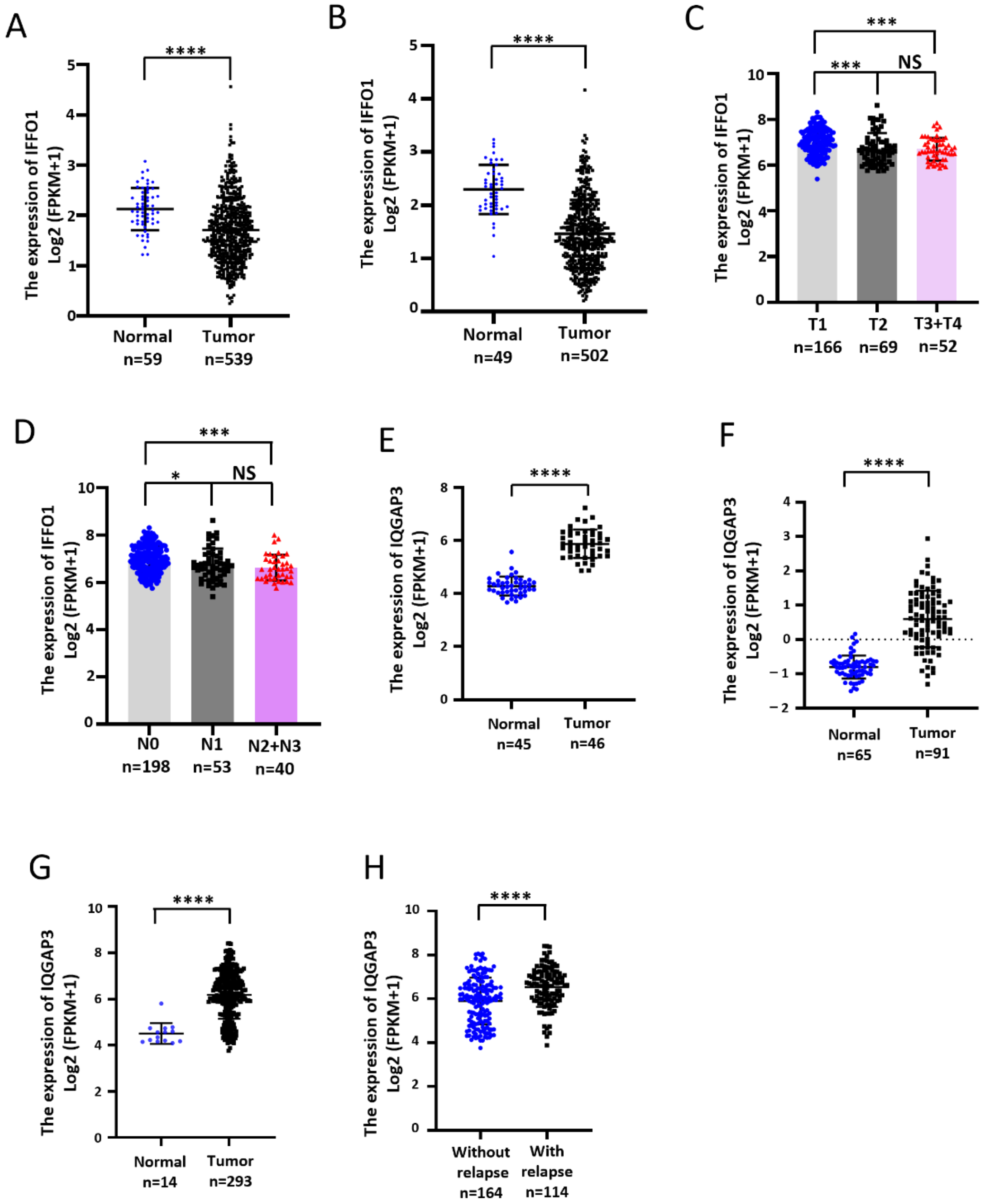

Supplementary Figure 7. Downregulation of IFFO1 in lung cancer is associated with tumor progression. IFFO1 expression between tumor tissues and normal tissues in the TCGA lung adenocarcinoma (LUAD) dataset (A) and TCGA lung squamous cell carcinoma (LUSC) dataset (B). Comparison of IFFO1 expression between T stages (C) and N stages (D) in the data obtained from the GSE30219 dataset. (E) Microarray analysis of IQGAP3 expression between tumor tissues and normal tissues from the GSE18842 dataset. (F) IQGAP3 expression profile between tumor tissues and adjacent normal tissues from the GSE19188 dataset. (G) Comparison of IQGAP3 expression between tumor samples and non-tumor lung samples from the GSE30219 dataset. (H) Comparison of IQGAP3 expression between tumor samples with or without relapse in the GSE30219 dataset. *$p < 0.05$. ***$p < 0.001$. ****$p < 0.0001$. NS, not significant.

Supplementary Table 1. Clinical information of patients

Patient	Age	Gender	Tumor size (cm)	Lymph node metastasis
1	57	Female	3.0	0/9
2	61	Female	5.0	0/5
3	59	Female	3.0	0/22
4	66	Male	3.0	0/9
5	68	Male	5.0	0/11
6	67	Male	5.5	0/20
7	53	Male	3.2	0/29
8	67	Female	2.5	0/7
9	62	Female	4.5	0/11
10	72	Male	2.3	0/8
11	71	Female	4.0	13/31
12	52	Male	4.5	12/18
13	52	Female	3.0	17/28
14	60	Male	2.5	2/12
15	49	Female	3.0	6/24
16	80	Female	2.5	15/28
17	66	Male	4.0	3/9
18	68	Female	4.0	5/9
19	66	Female	2.1	6/21
20	69	Male	4.5	2/13

Supplementary materials
Full and uncropped western blots

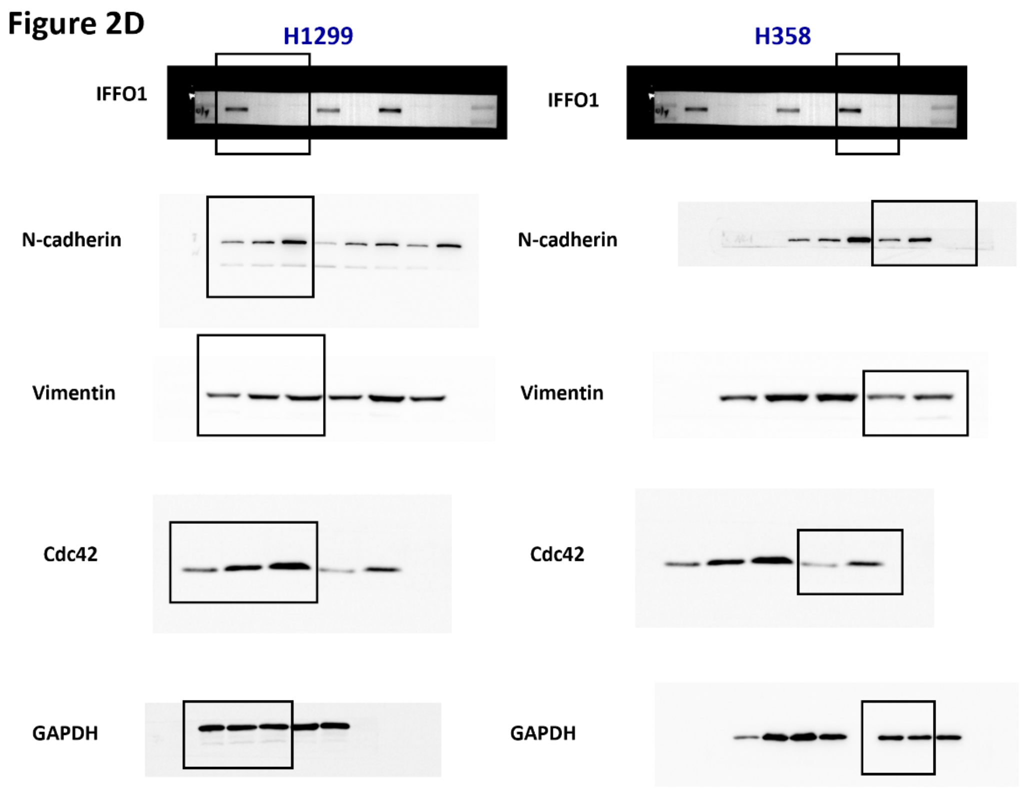

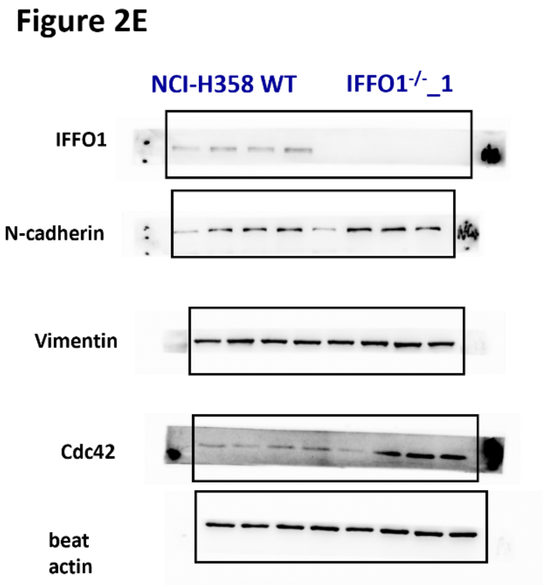

Figure 3B

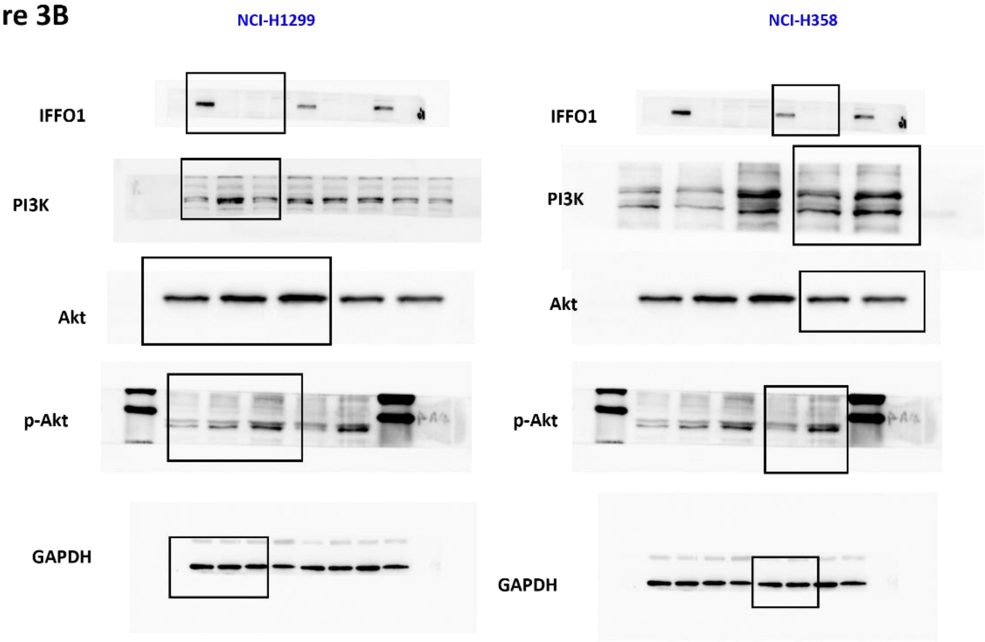

Figure 3C

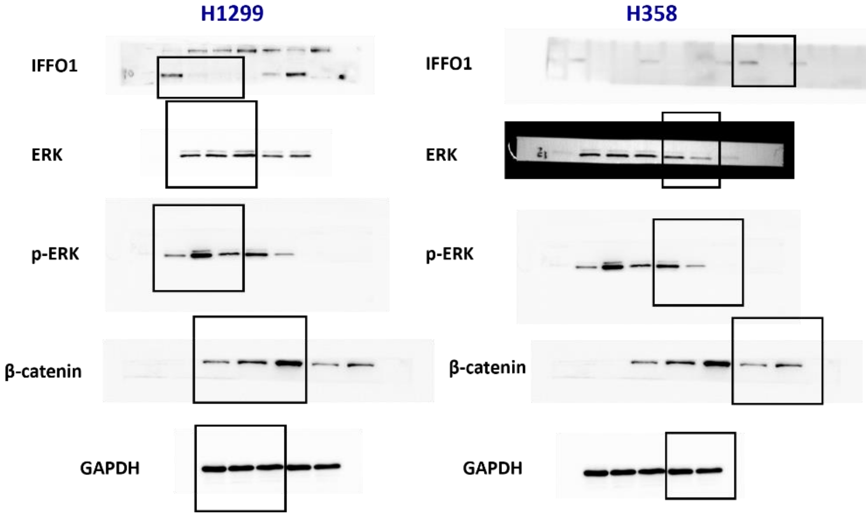

Figure 3G

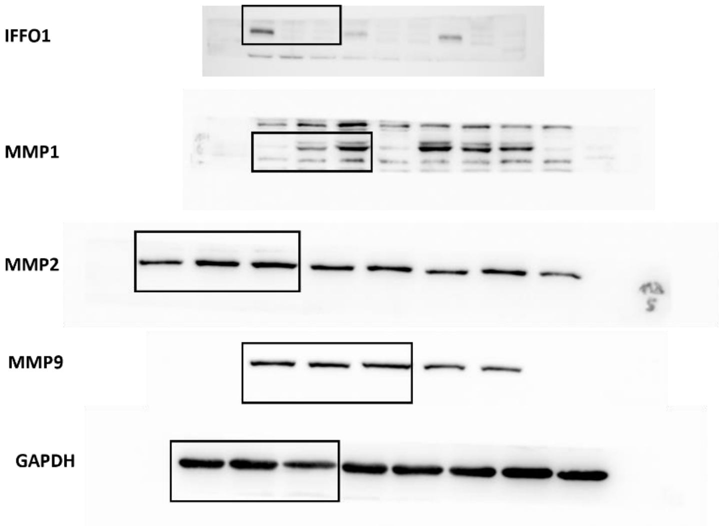

Figure 4B

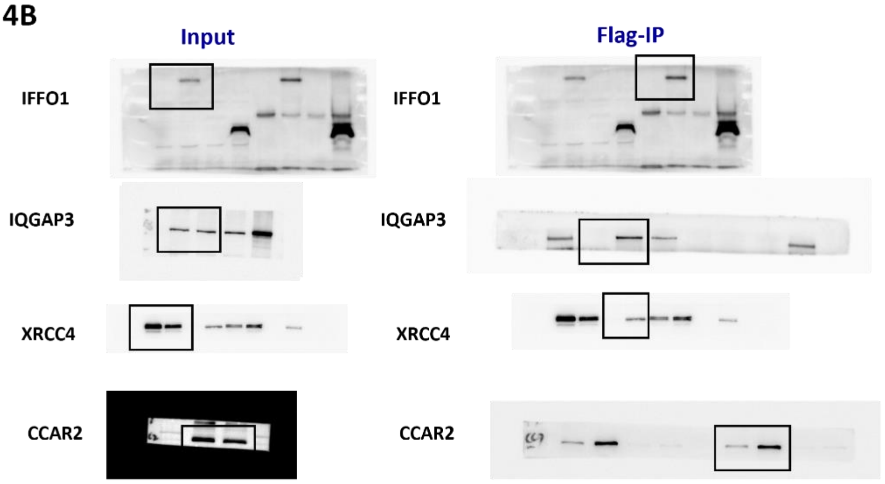

Figure 4C

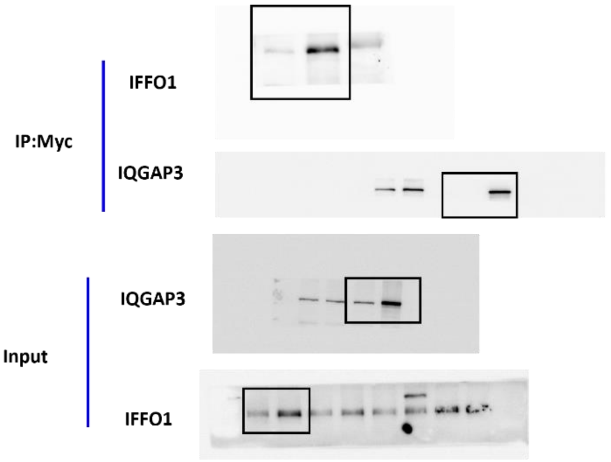

Figure 4D

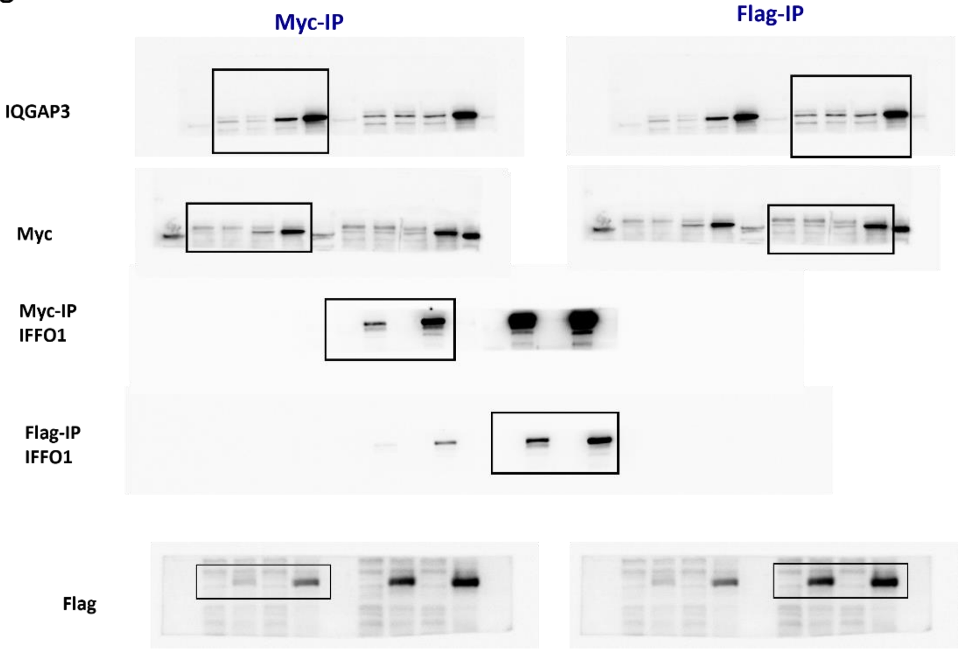

Figure 4F

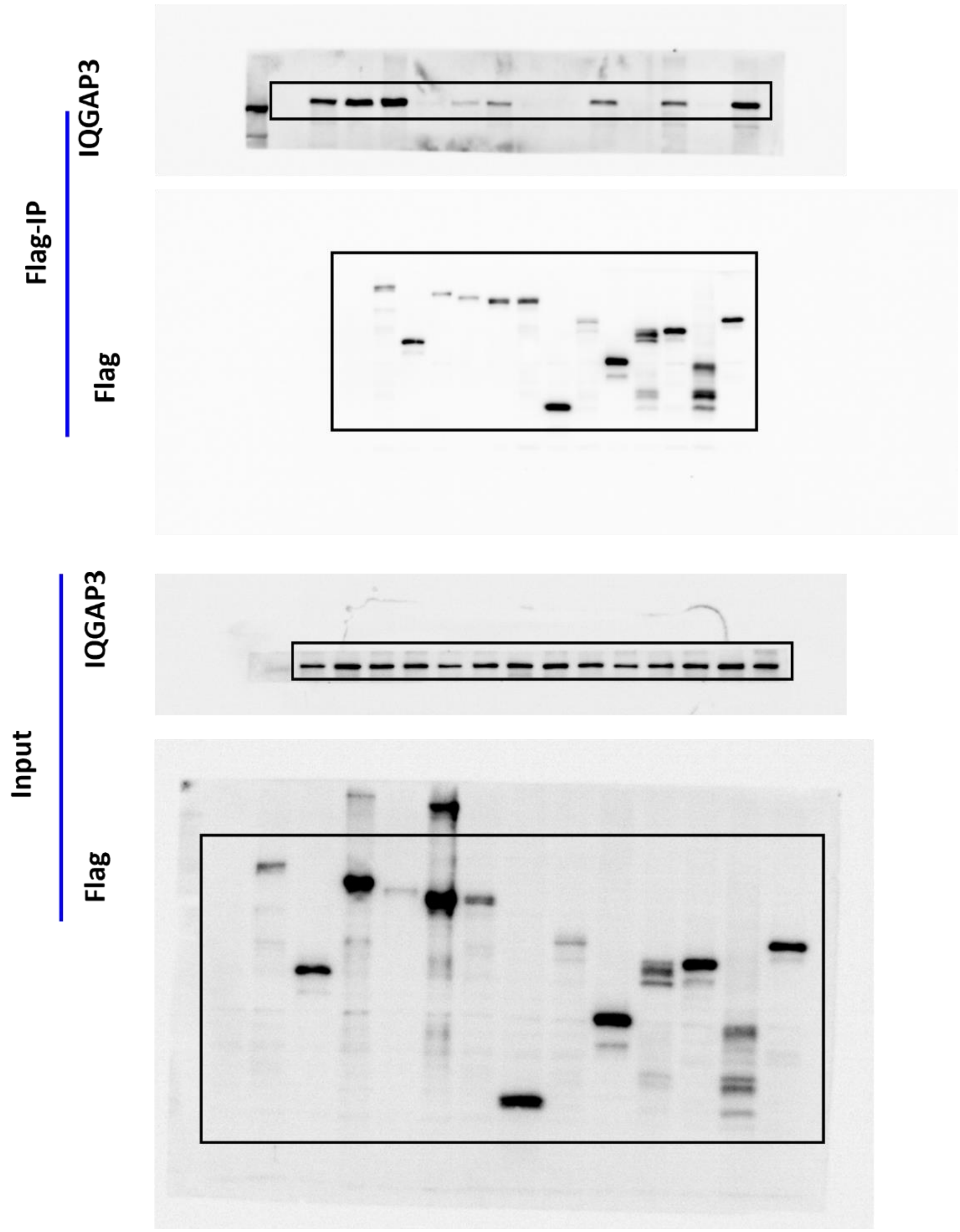

Figure 5A

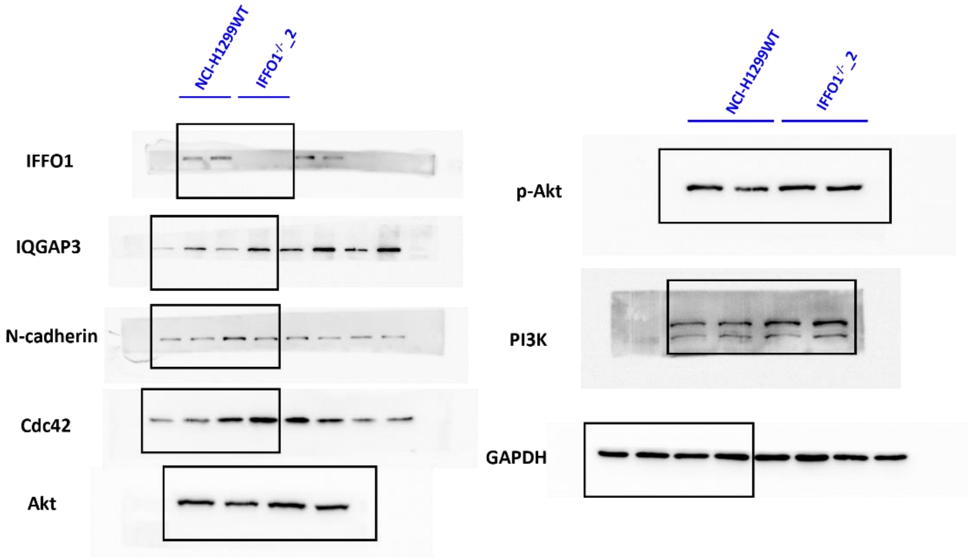

Figure 5B

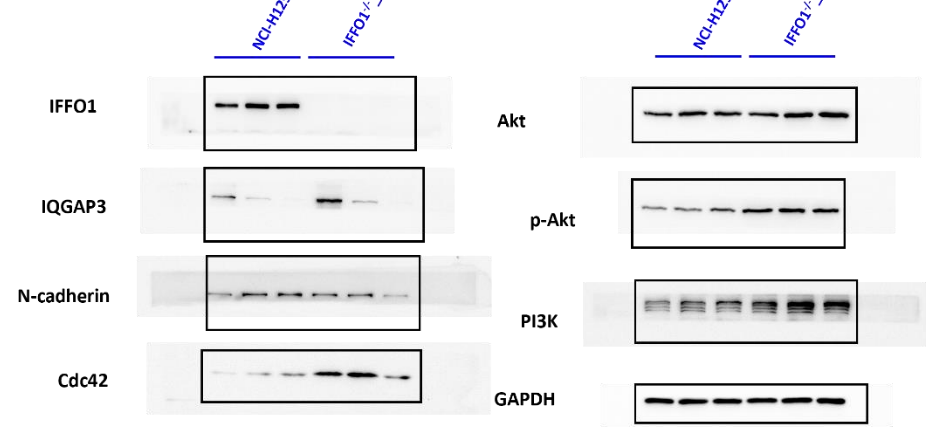

Figure 5F

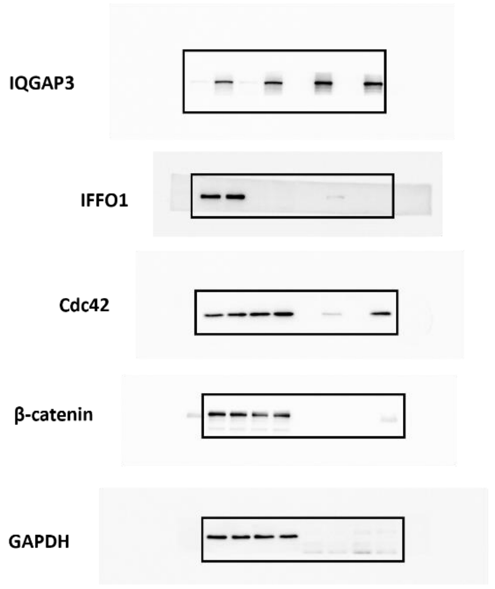

Figure 5G

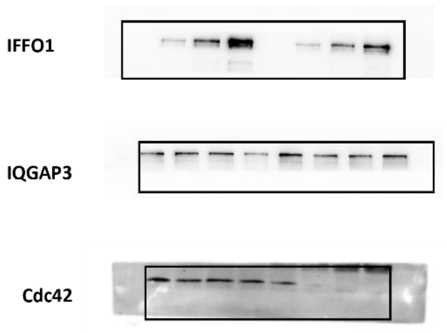

Figure 6C

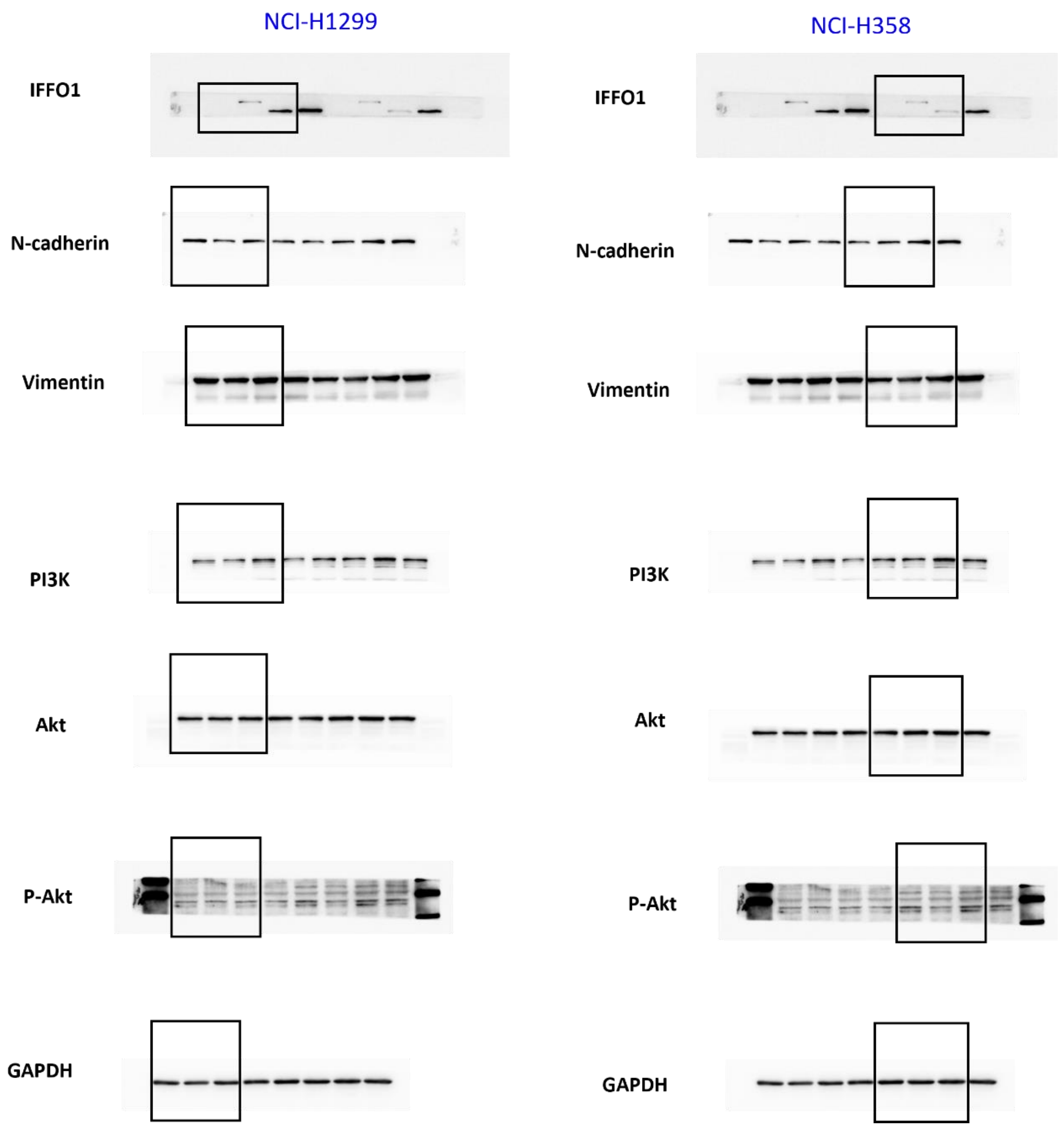
NCI-H1299
NCI-H358
IFFO1
N-cadherin
Vimentin
PI3K
Akt
P-Akt
GAPDH
IFFO1
N-cadherin
Vimentin
PI3K
Akt
P-Akt
GAPDH

Supplementary Figure 1A

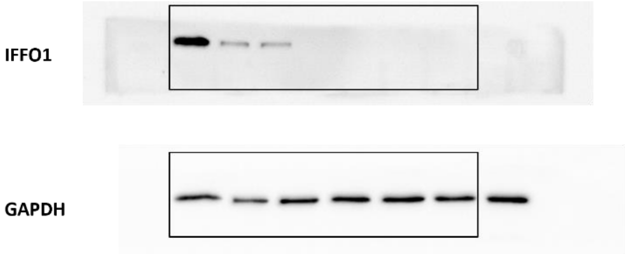

Supplementary Figure 1B

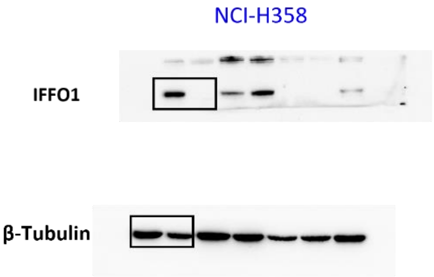

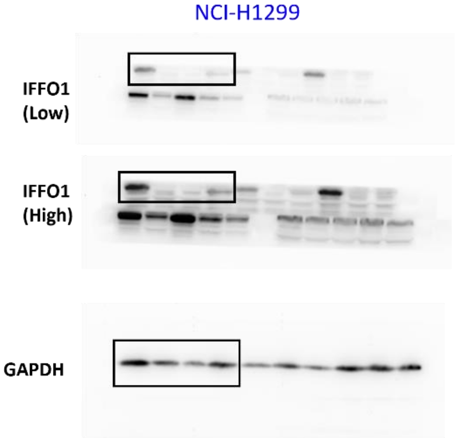

Supplementary Figure 1E

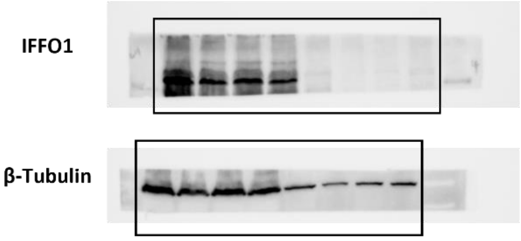

Supplementary Figure 1G

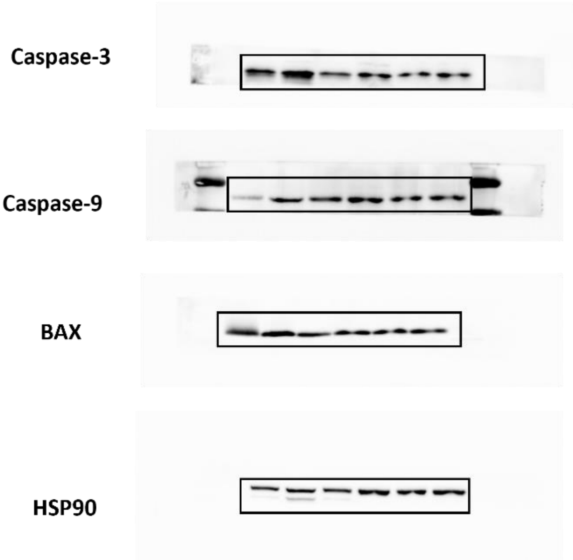

Supplementary Figure 2A

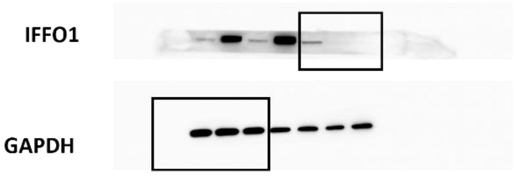

Supplementary Figure 2B

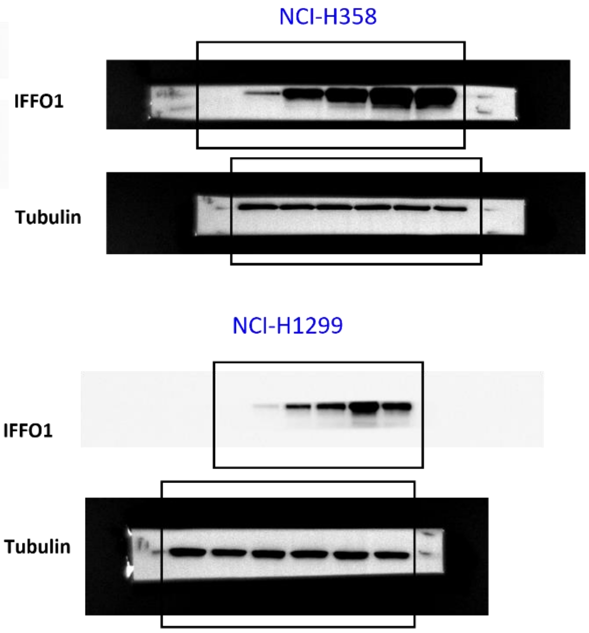

Supplementary Figure 2D

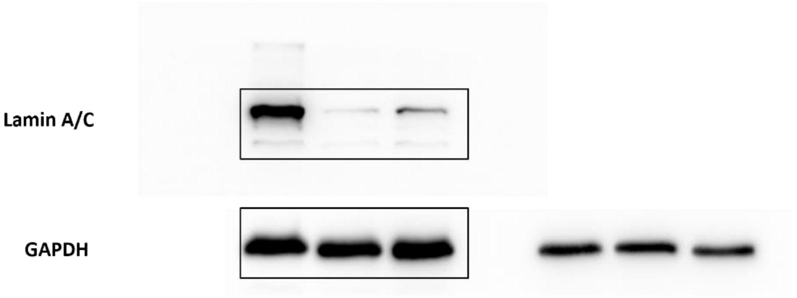

Supplementary Figure 4A

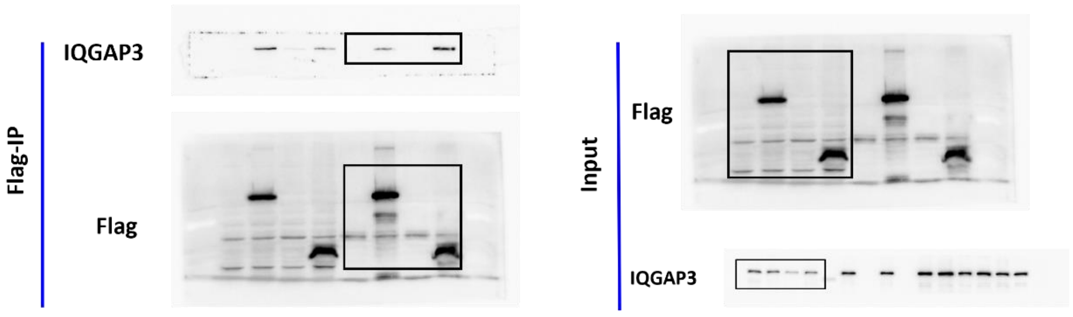

Supplementary Figure 4B

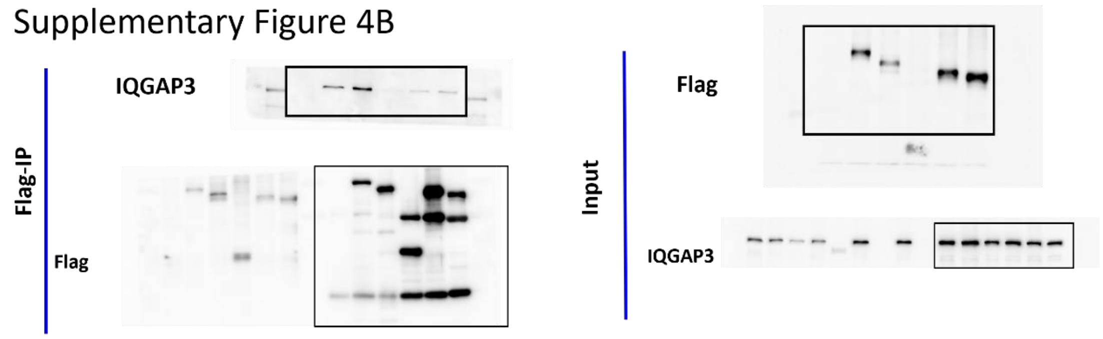

Supplementary Figure 4C

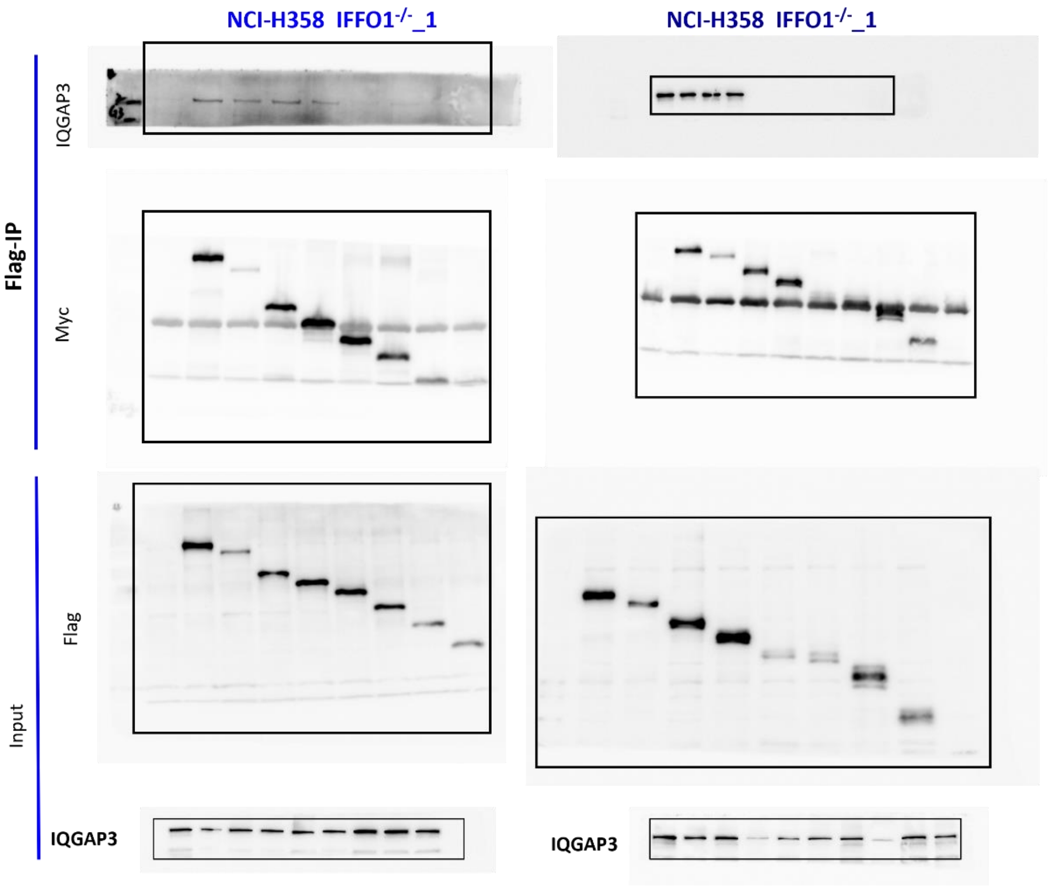

Supplementary Figure 4D

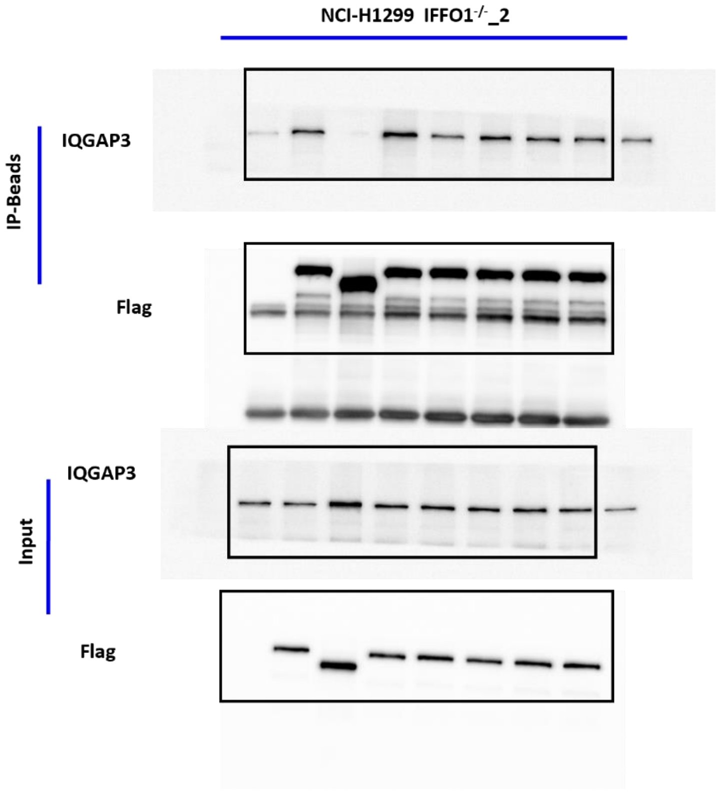

Supplementary Figure 5A

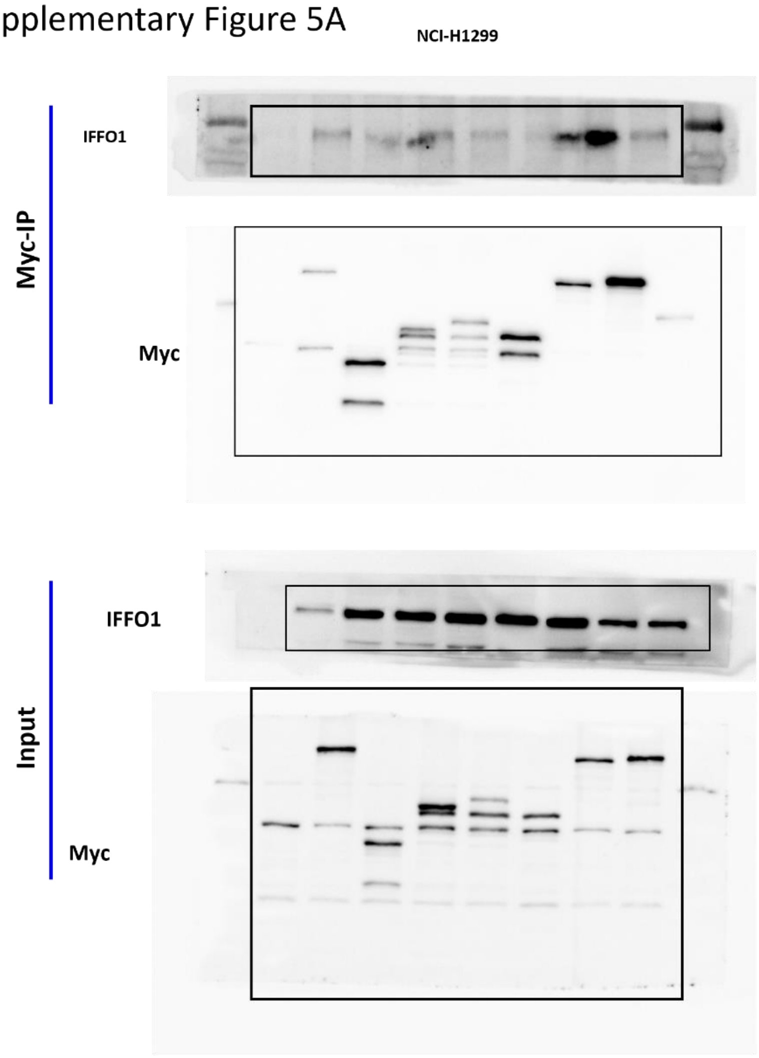

Supplementary Figure 5B

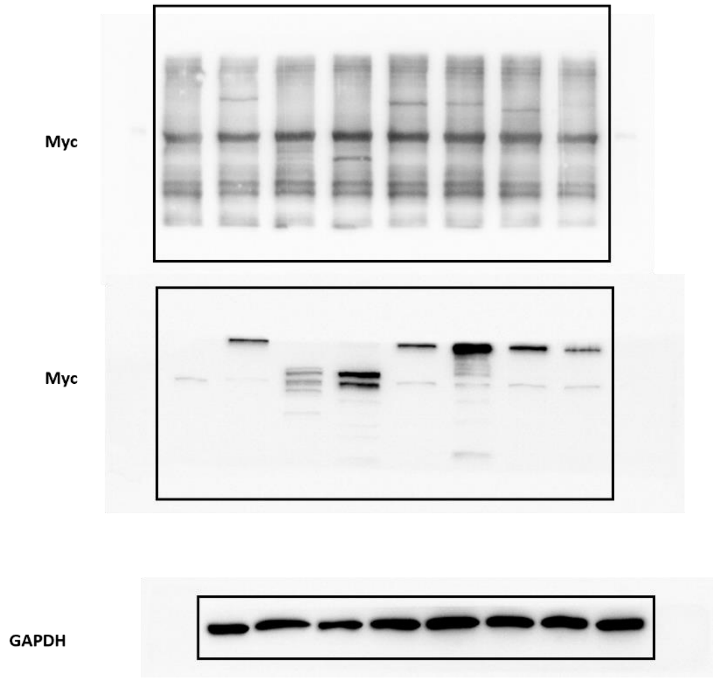

Supplementary Figure 6B

RAS

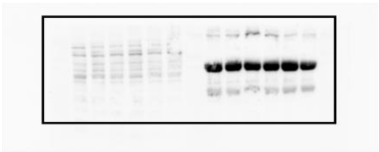

IQGAP3

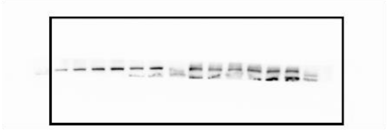

IFFO1

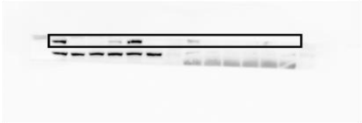

GAPDH

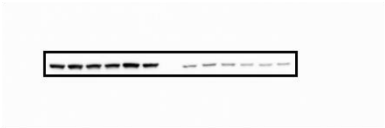

Supplementary Figure 6C

Flag

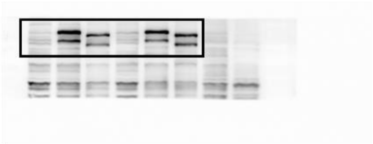

GAPDH

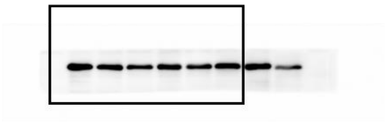

Supplementary Figure 6E

RAS

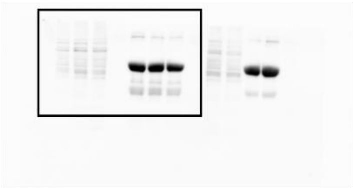

IFFO1

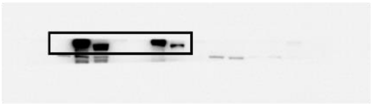

IQGAP3

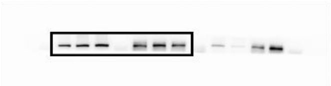

GAPDH

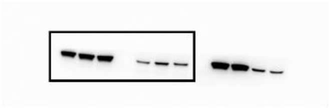

Supplement: Supplementary file 1 — Supplementary materials [file 41419_2025_7846_MOESM1_ESM.pdf]
